# Structural insights into dimethylation of 12S rRNA by TFB1M: indispensable role in translation of mitochondrial genes and mitochondrial function

**DOI:** 10.1093/nar/gkz505

**Published:** 2019-06-28

**Authors:** Xiaodan Liu, Shengqi Shen, Pengzhi Wu, Fudong Li, Xing Liu, Chongyuan Wang, Qingguo Gong, Jihui Wu, Xuebiao Yao, Huafeng Zhang, Yunyu Shi

**Affiliations:** 1 School of Life Sciences, University of Science & Technology of China, Hefei 230027, China; 2 Hefei National Laboratory for Physical Sciences at the Microscale, University of Science & Technology of China, Hefei 230027, China

## Abstract

Mitochondria are essential molecular machinery for the maintenance of cellular energy supply by the oxidative phosphorylation system (OXPHOS). Mitochondrial transcription factor B1 (TFB1M) is a dimethyltransferase that maintains mitochondrial homeostasis by catalyzing dimethylation of two adjacent adenines located in helix45 (h45) of 12S rRNA. This m^6^_2_A modification is indispensable for the assembly and maturation of human mitochondrial ribosomes. However, both the mechanism of TFB1M catalysis and the precise function of TFB1M in mitochondrial homeostasis are unknown. Here we report the crystal structures of a ternary complex of human (hs) TFB1M–h45–S-adenosyl-methionine and a binary complex hsTFB1M–h45. The structures revealed a distinct mode of hsTFB1M interaction with its rRNA substrate and with the initial enzymatic state involved in m^6^_2_A modification. The suppression of hsTFB1M protein level or the overexpression of inactive hsTFB1M mutants resulted in decreased ATP production and reduced expression of components of the mitochondrial OXPHOS without affecting transcription of the corresponding genes and their localization to the mitochondria. Therefore, hsTFB1M regulated the translation of mitochondrial genes rather than their transcription via m^6^_2_A modification in h45.

## INTRODUCTION

As a unique organelle in eukaryotes, mitochondria have retained a unique double-stranded circular DNA genome (mtDNA). The mtDNA genome encodes 22 transfer RNAs (tRNAs), two ribosomal RNAs (rRNAs), and 13 protein components of the oxidative phosphorylation system (OXPHOS), which produces the majority of cellular ATP (1–3). In addition, the expression of a substantial number of proteins and nucleic acids are coordinated by the mtDNA and nuclear genome to partake in respiration. OXPHOS complexes comprise 13 mtDNA-encoded subunits and 70 nuclear genome-encoded subunits that assemble to form four of the five enzymatic complexes essential for ATP production (4). Therefore, mitochondria play a vital role in eukaryotic energy conversion and metabolism. A growing number of diseases are considered to be inseparably linked to mitochondrial dysfunction, including age-related chronic diseases, inherited diseases and cancers (5,6).

Transcription of human mtDNA is initiated by unique molecular machinery in mitochondria, which contain a single-subunit RNA polymerase (mtRNAP), the mitochondrial transcription factor A (TFAM), and mitochondrial transcription factor B (TFBM). In mammalian mitochondria, two TFBMs (TFB1M and TFB2M) are synthesized as bifunctional proteins functioning as transcription factors and methyltransferases. TFB2M is the primary mitochondrial transcription initiation factor and is a component of the mitochondrial transcription initiation complex. TFB1M was originally thought to serve as a transcription factor along with TFB2M but has instead been shown to be a dimethyltransferase with the main role of m^6^_2_A modification of the 3′ end of mitochondrial 12S rRNA (7–9). The sequence and structure of a stem loop [helix 45 (h45)] located in the terminal helix of 12S rRNA is conserved across all three domains (Supplementary Figure S1A). Two nucleotides near the 3′ end of the stem loop, m. 936A and m. 937A, can be m^6^_2_A-dimethylated by TFB1M. This is the only example of RNA post-transcriptional modification by dimethylation found in the cell till now (9–12).

In the wake of extensive research in epigenetics, RNA post-transcriptional modifications have gradually been known to regulate various cellular processes. More than 100 different kinds of RNA modifications have been identified in tRNAs, rRNAs and messenger RNAs (mRNAs), as well as in other types of non-coding RNA (2,13). In mitochondria, modifications to rRNA are essential to maintain the stability and functionality of the core translation machinery, to regulate RNA fate in metabolic pathways, and to control gene expression at the level of translation (2,14–16). Human mitochondrial 12S rRNA is located in the mitochondrial small 28S subunit (mt-SSU), and together with several proteins and 16S rRNA in the mitochondrial large 39S subunit (mt-LSU), it constitutes the 55S mitochondrial ribosome. Ribosome biogenesis is vitally important for mitochondrial gene expression. Multiple factors affect ribosome assembly, and rRNA post-transcriptional modifications can directly impact transcript stability, polyadenylation profile and processing, ribosome biogenesis, and perhaps other elements in eukaryotic cells (17–21). Metodiev *et al.* demonstrated that methylation of 12S rRNA was necessary for the *in vivo* stability of the mtSSU, and that dimethylation was a potential checkpoint or a precondition for ribosome assembly; lower levels of dimethylation resulted in mtSSU assembly deficiency, negatively influencing translation efficiency (9).

The methyltransferases performing the m^6^_2_A modification are highly conserved from prokaryotes to eukaryotes. KsgA has been identified as a methyltransferase in prokaryotes, and it dimethylates 1518A and 1519A of 16S rRNA in *Escherichia coli* (22,23). The homologous eukaryotic proteins, including *Saccharomyces cerevisiae* Dim1 (24,25), *Homo sapiens* Dim1 (26), *Arabidopsis thaliana* Pfc1 (27) and *Homo sapiens* TFBM (28), are all responsible for the dimethylation of two adjacent adenines near the termini of rRNAs. Although the crystal structures of prokaryotic methyltransferases, as well as a complex between mouse TFB1M and S-adenosyl-methionine (SAM), have been determined (29), the detailed mechanism of m^6^_2_A modification cannot truly be understood without structural information on the enzyme and its substrate rRNA, particularly in mammals. Cramer *et al.* explained the mechanism of transcription initiation based on a structure of the mitochondrial transcription initiation complex containing mtRNAP, TFAM, TFB2M, and promoter DNA (30). However, TFB1M was absent in this complex. In the last decade, metabolism studies on the relation between TFB1M and type 2 diabetes (T2D) have been gradually deepening. Mining the DGI GWAS for genes involved in OXPHOS led to the identification of a common variant (rs950994) located in intron 2 of the human *TFB1M* gene, and subsequently analysis of a prospective cohort revealed a pathogenetic effect of the risk variant in individuals carrying an A-allele, which could predict the occurrence of T2D in future. Further, the islets of TFB1M-deficient mice displayed mitochondrial dysfunction and released less insulin in response to glucose, likely contributing to the pathogenesis of T2D (31–33). However, the overexpression of TFB1M in the mouse showed no impact on mitoribosomal methylation status or hearing (34). Moreover, homozygous *Tfb1m^−/−^*knockout mice showed a lethal embryonic phenotype (9). Given its roles in ribosome biogenesis and protein translation, TFB1M has been implicated in other diseases associated with mitochondrial dysfunction, such as cancer (35,36). Nevertheless, we still do not have a full understanding of how TFB1M works in mitochondria or how m^6^_2_A modification regulates mitochondrial gene expression.

To clarify the mechanism of m^6^_2_A modification, we determined the crystal structures of a ternary complex of human (hs) TFB1M (hsTFB1M–h45–SAM) and a binary complex (hsTFB1M–h45) both at 3.0 Å resolution. In the structures, the ‘GGAA’ tetraloop of 12S h45 flipped out to interact with hsTFB1M. The terminal adenosine in the ‘GGAA’ tetraloop, m. 937A, was favored for modification by hsTFB1M because it entered the enzyme active site in the initial state. We further identified residues located in the enzyme active site and the interface between hsTFB1M and h45 using binding affinity and enzyme activity assays. We also performed nuclear magnetic resonance (NMR) experiments to compare the solution structures of h45 with (m^6^_2_A-h45) or without (h45) dimethylation.

Moreover, the knockdown of hsTFB1M in hepatocellular carcinoma cells, as well as the mutation of key residues, impaired ATP production and reduced the expression of several protein components of the mitochondrial OXPHOS, but did not affect transcription of the corresponding genes (key factors in complexes I–IV of the respiratory chain). Together, these data showed that hsTFB1M plays an essential role in m^6^_2_A dimethylation that may be relevant for the assembly of mitochondrial ribosomes, thereby regulating the translation rather than the transcription of mitochondrial genes and playing an indispensable role in mitochondrial function.

## MATERIALS AND METHODS

### Cloning, expression, and purification of hsTFB1M

The hsTFB1M open reading frame (residues 28–346; UniProt Q8WVM0) was amplified using PCR from a cDNA library constructed from the human brain and spinal cord tissue using the following oligonucleotide primers: 5′-CGCGGATCCATGCAAGCAGCGAAGCAGCTATCACAG-3′ (hsTFB1M_forward) and 5′-CCGCTCGAGCTAGAGTCTGTAATTCTCTGCGTCATC-3′ (hsTFB1M_reverse). The amplified fragment was cloned into the modified pET 27b vector, which contained N-terminal His_6_ and maltose-binding protein (MBP) tags. The His_6_-tagged MBP-hsTFB1M fusion protein was expressed in *E. coli* strain BL21-Gold (DE3) pLysS cells. The cells were grown to mid-log phase (OD, 0.8–1.0) in Luria broth containing 50 μg/mL kanamycin at 37°C. The overexpression of the fusion protein was induced using 1 mM isopropyl-β-D-thiogalactopyranoside for 24 h at 16°C, after which the cells were collected by centrifugation.

Cells paste from 1 l of culture was suspended in 40 ml of pre-cooled buffer A (50 mM sodium phosphate, pH 7.5, containing 1 M sodium chloride). A FB-110X homogenizer was used to lyse the cells at a pressure of 800 bar. The cells were subsequently lysed by sonication on ice to remove nonspecific-binding nucleic acids and centrifuged at 13 000 × g for 30 min. The protein-containing supernatant was subjected to affinity chromatography using a Ni^2+^-chelating column (GE Healthcare), eluted with pre-cooled buffer B (50 mM sodium phosphate, pH 7.5, containing 1 M sodium chloride and 0.5 M imidazole) and purified by size-exclusion chromatography using a Superdex 200 (GE Healthcare) column. The purified fusion protein was digested overnight at 16°C with His_6_-tagged TEV protease; next, cleaved MBP and residual TEV protease were removed using a Ni^2+^-chelating column. The purified hsTFB1M protein was diluted in buffer C (50 mM sodium phosphate, pH 7.5, containing 200 mM NaCl).

### Crystallization, data collection and structure determination

HsTFB1M was concentrated to 10 mg/ml in buffer C and mixed with unmodified h45 at a 1:1.2 molar ratio for the binary complex and mixed with S-adenosyl-l-methionine (AdoMet, SAM) (Sigma) at a 1:1.2:3 molar ratio for the ternary complex crystallization. The hsTFB1M–h45 complex crystal was grown at 293 K by using the sessile drop method with the mother liquor, pH 5.0, containing 0.1 M sodium citrate and 20% PEG8000, and the hsTFB1M–h45–SAM complex crystal was grown with mother liquor, pH 7.0, containing 0.2 M potassium chloride, 0.025 M magnesium sulfate hydrate, 0.05 M HEPES, and 20% (v/v) polyethylene glycol 200. The crystals were soaked in the mother liquor supplemented with 25% (v/v) glycerol and flash-frozen in liquid nitrogen. X-ray diffraction data were collected using beamline 19U1 of the Shanghai Synchrotron Radiation Facility (SSRF). The data were manipulated using the HKL2000 software (37). The structures of the complex were determined by molecular replacement with the MOLREP program (38) using the structure of murine TFB1M (PDB ID: 4GC5) as the search model. The REFMACS5 (39) and COOT (40) programs were used to build and refine the models further, and *R*_work_ and *R*_free_ were refined to 0.22 and 0.26 for hsTFB1M–h45–SAM and 0.19 and 0.24 for hsTFB1M–h45, respectively. Data collection and processing statistics are summarized in Supplementary Table S3.

### Electrophoresis mobility shift assay (EMSA)

EMSA was used to measure the binding affinities of wild-type (WT) and mutant hsTFB1M for h45 (RNA sequences shown in Supplementary Table S1). Lyophilized 5′-FAM-labeled RNA oligomers were purchased from Takara (Takara Biomedical Technology Co., Ltd) and dissolved in diethylpyrocarbonate (DEPC)-treated water at a concentration of 10 μM. Experiments were performed in 10 μl volumes of buffer D (50 mM Tris–HCl, pH 8.0, containing 25 mM sodium chloride) containing 100 nM RNA and increasing concentrations of TFB1M (0–2 mM). Reactions were incubated at 37°C for 30 min and then resolved on non-denaturing 6% 19:1 polyacrylamide gels (0.5× Tris-borate/EDTA running buffer, 40% sucrose loading buffer) at 120 V for 20 min. Gels were scanned using a Typhoon FLA 7000 instrument (GE Healthcare). Each experiment was performed in triplicate.

### Glutathione S-transferase (GST) pull-down assay

cDNAs encoding hsTFB1M or its mutants were cloned into the pGEX4T1 vector (Novagen) using the ClonExpressTM II One Step Cloning Kit (Vazyme). Proteins were produced in *E. coli* BL21 (DE3) cells. Purified His_6_- and GST-tagged proteins were incubated in pull-down buffer [50 mM Tris, pH 7.5, containing 150 mM NaCl, 0.1% NP-40 and 5 mM dithiothreitol (DTT)]. After incubation, beads were pelleted and washed with washing buffer [8 mM Na_2_HPO_4_, 136 mM NaCl, 2 mM KH_2_PO_4_, 2.6 mM KCl, 0.05% (V/V) Tween-20, pH 7.2–7.4] and then used for immunoprecipitation and primer extension analysis.

### *In vitro* methyltransferase activity assay

Radioisotope labeling and primer extension assays were both used to assess the *in vitro* activity of hsTFB1M. For radiolabeling, each 40-μl reaction contained 50 mM Tris–HCl, pH 8.0, 1 mM spermidine, 3 mM MgCl_2_, 1 mM DTT, 50 mM NH_4_Ac, 2 μCi ^3^H-SAM (or 5 mM SAM), 0.5 μM hsTFB1M (overexpressed in *E. coli* or purified from GST pull-down experiments) and 3.2 μM h45 RNA. The mixture was incubated at 30°C for 2 h; then, 100 μl of water-saturated phenol was added to stop the reaction. The mixture was centrifuged to separate the aqueous phase from the phenolic phase. An equal volume of chloroform/isoamyl alcohol (24:1 v/v) was added to the aqueous phase for further extraction, after which h45 RNA was precipitated at −20°C through the addition of three volumes of pre-cooled ethanol. The precipitates were collected by filtration through a nitrocellulose membrane (Millipore), washed with pre-cooled ethanol, and air-dried for scintillation counting. For primer extension, the precipitates were air-dried at room temperature and dissolved in DEPC-treated water.

### Primer extension analysis

Primer extension analysis was changed based on reference (25). A 5′-Cy5-labeled DNA primer (16 nucleotides: 5′-CTGGTTCGTCCAAGTG-3′) complementary to a sequence downstream of the 3′ end of 12S rRNA was synthesized by Sangon Biotech (China). Target RNA (4 μg) produced from methyltransferase activity experiments was incubated with 50 pmol of 5′-Cy5-labeled primer at 65°C for 20 min and then on ice for at least 3 min. Primers were extended in 20-μl reactions containing 200 units of SuperScript III Reverse Transcriptase (Invitrogen), 5× RT buffer, 1 μl of 0.1 M DTT, and 250 μM dNTP mix (containing ddGTP in place of dGTP) and incubated at 50°C for 10 min, 95°C for 2 min, and at 4°C until analyses. Extension products were resolved on 18% denaturing urea-PAGE gels with 80% formamide loading buffer and scanned using a Typhoon FLA 7000 instrument (GE Healthcare). Each experiment was performed in triplicate.

### Cell lines

PLC and HEK293T cells were cultured in Dulbecco's modified Eagle's medium supplemented with 10% (v/v) fetal bovine serum and 1% penicillin/streptomycin. Cells were grown at 37°C in a humidified atmosphere containing 5% CO_2_. Trypan blue exclusion was used to assess cell viability.

### Western blotting

Cell lysates were prepared in radioimmunoprecipitation assay buffer (50 mM Tris–HCl, pH 8.0, containing 150 mM NaCl, 5 mM EDTA, 0.1% SDS, and 1% NP-40) supplemented with protease inhibitor cocktails (Roche). Equal amounts of total protein were separated by SDS-PAGE. Primary antibodies against the following proteins were used in western blotting: TFB1M (A15231, abclonal), MT-CO1 (55071-1-AP, PTG), MT-CO2 (55070-1-AP, PTG), MT-CYB (55090-1-AP, PTG), MT-ND1 (19703-1-AP, PTG), MT-ND5 (55410-1-AP, PTG), MT-ATP8 (26723-1-AP, PTG), MRPS34 (15166-1-AP, PTG), MRPS16 (16735-1-AP, PTG), MRPS15(17006-1-AP, PTG), MRPL13 (16241-1-AP, PTG), MRPL48 (14677-1-AP, PTG), anti-puromycin (MABE343). β-Actin (66009-1-Ig, Abmart) and COX4 (11242-1-AP, PTG) served as a loading control. Horseradish peroxidase-conjugated anti-rabbit and anti-mouse (Bio-Rad) secondary antibodies were used to detect primary antibody binding and signal was detected using Western ECL substrate (Bio-Rad).

### Immunoprecipitation (IP) assay

Cells were lysed with high-salt IP buffer (20 mM HEPES, pH 7.5, containing 0.5% NP-40, 300 mM NaCl, 2 mM EDTA and 1.5 mM MgCl_2_) supplemented with a protease inhibitor cocktail for 1–2 h on ice, then centrifuged at 12 000 × g for 10 min at 4°C. The supernatants were incubated with GST beads loaded with TFB1M or TFB1M mutants overnight at 4°C. The beads were then washed with IP buffer and stored in IP buffer supplemented with protease inhibitors until further analysis.

### Immunofluorescence

HepG2 cells were used for immunofluorescence experiments. Cells were transfected with TFB1M plasmids (WT and mutants E85A, K86A, D111A, V112A, R183E/R256E/R257E were cloned into p3 × Flag-cmyc vectors) using Lipofectamine 3000. Twenty four hours after the transfection, cells were stained with 500 nM Mitotracker (Invitrogen) for 40 min in an incubator. Cells were fixed in freshly prepared 3.7% paraformaldehyde in PBS for 15 min at RT followed by permeabilization with 0.2% Triton X-100 in PBS for 5 min and blocked with 1% bovine serum albumin in PBS with 0.3% Triton. These cells were incubated with the Myc-tag antibody (Cell Signaling, mouse antibody) in a humidified chamber overnight at 4°C and washed three times with TPBS (0.05% Tween-20 in PBS). Primary antibodies were visualized with FITC-conjugated goat anti-mouse IgG (Jackson ImmunoResearch). DNA was stained with DAPI (Sigma).

### Plasmids and stable cell lines

All short hairpin RNAs (shRNAs) (except sh3′ UTR) against TFB1M were purchased commercially in the pLKO vector (Sigma-Aldrich). The shRNA targeting the TFB1M 3′ UTR was designed and subcloned into the pLKO vector. Genes encoding TFB1M and TFB1M mutants were subcloned into the pSin-3 × Flag vector. PLC cells were infected with lentivirus followed by antibiotic selection to establish stable cell lines.

### Cellular ATP measurement

ATP levels were determined using as luciferin–luciferase-based ATP assay kit (Promega) and a luminescence reader (Promega). Luminescence was normalized to protein concentrations.

### Mitochondrial isolation

PLC cells were suspended in ice cold buffer containing 10 mM sucrose, 50 mM Tris–HCl (pH 7.4), 100 mM KCl, 1.5 mM MgCl_2_, 1 mM EGTA, 50 mM HEPES, 100 μg/ml chloramphenicol and 100 μg/ml thiamphenicol and dissociated using a MACS Dissociator (Miltenyi Biotech) with the human mitochondria isolation program. The supernatant containing the mitochondria was obtained by centrifugation of the samples twice for 10 min at 850 g to remove the nucleus. Mitochondria were pelleted by centrifugation for 30 min at 10 000 × g and washed twice in the same buffer.

### Sucrose gradient sedimentation

4 mg mitochondria from NTC- or shTFB1M-expressing PLC cells treated with 100μg/ mL Chloramphenicol & Thiamphenicol for 30 min was lysed with lysis buffer (260 mM sucrose, 100 mM KCl, 20 mM MgCl_2_, 10 mM Tris–Cl [pH 7.5], 1% Triton X-100, 5 mM β-mercaptoethanol, protease inhibitor cocktail without EDTA (Roche), 10 U/μl RNase inhibitor(BBI), 100 μg/ml Chloramphenicol & Thiamphenicol) on ice for 30 min. Lysate was precleared by centrifugation at 10 000 × g for 15 min at 4°C, then the lysates were loaded on a 4 ml 10–30% continuous sucrose gradient (10 mM Tris–Cl, 100 mM KCl, 20 mM MgCl_2,_ 5 mM β-mercaptoethanol, 10 μg/ml Chloramphenicol & Thiamphenicol, protease inhibitor cocktail without EDTA, 1 U/μl RNase inhibitor) and centrifuged at 180 000 ×g for 4 h in a Beckman MLS-50 rotor at 4°C. After centrifugation, gradients were fractionated from the top into 24 equal fractions. Half of each fraction was used for RNA isolation by TRIzol Reagent (Ambion). Isolated RNA was dissolved in 100 μl DEPC water and the RNA concentration was detected by spectrophotometer (One Drop). The other half of each fraction was mixed with adjacent half fraction together and then was subjected to western blot analysis using the anti-MRPS15, anti-MRPS16, anti-MRPS34, anti-MRPL13, anti-MRPL48 antibodies.

### SUnSET translation assays

For the SUnSET assays (41), non-targeting control (NTC) cells and TFB1M-depleted stable PLC cells (selected by hygromycin) were seeded in culture dishes 8 h prior to serum starvation. For serum stimulation, cells were maintained in DMEM containing 10% fetal bovine serum for 2 h and then exposed to 100× cycloheximide (CHX; 1 mg/ml) to inhibit the cytoplasmic ribosome for 20 min. Puromycin pulses were performed by incubating the cells with 10 mg/ml puromycin for 30 min at 37°C. Cells were then washed with cold PBS and lysed in RIPA buffer supplemented with 1 mM PMSF and protease inhibitor cocktail (Roche) to obtain the whole cell lysate. For the mitochondrial fraction, mitochondria were isolated from cells and lysed in the same buffer. Equal amounts of the lysates were analyzed by western blot analysis using the anti-puromycin antibody.

### OP-puromycin labeling of cultured cells and detection by fluorescence microscopy

For OP-puromycin assays (42), NTC cells and TFB1M-depleted stable PLC cells were grown in 96-well plates in DMEM containing 10% fetal bovine serum. 100× CHX (1 mg/ml) was added to cells in the culture medium, and the cells were incubated for 15 min. Finally, a Click-iT Plus OPP Protein Synthesis Assay Kit (Thermo Fish C10456) was applied to the cells as per the manufacturer's instructions. The stained cells were imaged by fluorescence microscopy (OLYMPUS IX51).

### Statistical analysis

The data were presented as means ± standard deviations or means ± standard errors of the means as stated. Differences between groups were evaluated using Student's *t*-tests. *P* values <0.05 were considered significant. Statistical significance is displayed in figures as ** for *P* < 0.01.

### Preparation and purification of 12S h45 RNA oligonucleotides

The 28-nucleotide unmodified h45 RNA (h45, 5′-GGUAAGUGUACUGGAAAGUGCACUUGCC-3′) was transcribed *in vitro* with T7 RNA polymerase, which added ‘GG’ and ‘CC’ to its 5′ and 3′ ends, respectively (Supplementary Figure S1A), for efficient transcription (43). The transcription reaction, containing 40 mM Tris–HCl, pH 8.1, 1 mM spermidine, 0.01% (v/v) Triton X-100, 10 mM DTT, 25 mM MgCl_2_, 0.1 μM single-stranded DNA template (5′-GGCAAGTGCACTTTCCAGTACACTTACCTATAGTGAGTCGTATTAATTTC-3′), 0.1 μM T7 promoter primer (5′-GAAATTAATACGACTCACTATAG-3′), 1 mg of T7 promoter, and 5 mM each of unlabeled or ^13^C- or ^15^N-labeled NTPs (SILANTES GmbH), was incubated for 4 h at 37°C. Target RNA was further purified by electrophoresis on large 12% denaturing urea-PAGE gels, electroeluted in 0.5× Tris-borate/EDTA buffer using the Elutrap Electroelution System (Whatman), then desalted and buffer exchanged extensively into NMR buffer (10 mM sodium phosphate, pH 6.5, containing 50 mM sodium chloride) using Amicon Ultra centrifugal filter devices (Millipore).

Modified h45 RNA (m^6^_2_A-h45, 5′-UAAGUGUACUGGm^6^_2_Am^6^_2_AAGUGCACUUG-3′) was purchased from Biomers (http://www.biomers.net, Germany) and exchanged into the same NMR buffer.

### Preparation of target RNA samples for NMR

The purified RNA was maintained in a H_2_O/D_2_O (90%/10%) mixture used for NMR experiments examining exchangeable protons. Samples prepared for non-exchangeable proton experiments in D_2_O were lyophilized twice. Deuterium oxide (D_2_O, 99.96%) was added separately. All NMR samples contained 1–1.5 mM RNA; RNA samples were refolded by heating at 95°C for 5 min, fast-cooling on ice for 20 min, and concentrating to smaller volumes.

### NMR spectroscopy

All NMR experiments were performed on a Bruker Avance 600 MHz spectrometer equipped with HCN cryoprobes. The temperatures of the experiments performed in H_2_O and D_2_O were 283 and 303 K, respectively. NMRPipe (24) and Sparky (44) (https://www.cgl.ucsf.edu/home/sparky) were used for spectrum assignment and analysis.

The 2D NOESY (mixing time: 200 ms) spectrum for unmodified h45 in H_2_O, together with the 2D ^1^H–^15^N HNN–COSY, ^1^H–^15^N HSQC and H6/H5(C4N)H spectra were initially analyzed to assign imino and amino exchangeable protons. Assignments for all non-exchangeable protons were obtained from 2D NOESY in D_2_O, as well as 2D TOCSY, 2D MQ-HCN, 2D ^1^H–^13^C CT-HSQC, 3D ^1^H–^13^C NOESY-HSQC and 3D HCCH-TOCSY spectra. The resonances of base protons (H8/H6) and sugar protons (H1′) were analyzed in the 2D NOESY spectrum in D_2_O. The sugar pucker was determined with 2D DQF-COSY. Assignments for m^6^_2_A-h45 were mainly obtained from 2D NOESY spectra in H_2_O and D_2_O, and together with the 2D DQF-COSY and the TOCSY spectra, compared with the spectra of unmodified h45.

### NMR structure calculations and refinements

The backbone dihedral angles of residues in the stem region, including α, β, γ, δ, ϵ and ζ, were constrained to the A-form RNA helix geometry (α = −87.0° ± 50°, β = −205.0° ± 50°, γ = 23.0° ± 50°, δ = 58.0° ± 50°, ϵ = −177.0° ± 50°, and ζ = −98.0° ± 50°). No constraints were applied to the GGAA tetraloop. Interproton distance restraints were derived according to 2D NOESY spectra. Upper distance limits of 2.7, 3.3, 5.0 and 6.0 Å were generally employed for cross-peaks of strong, medium, weak and very weak intensity, respectively.

Initial structures of h45 and m^6^_2_A-h45 were produced with CYANA 3.0 (45) wherein parameters for m^6^_2_A of dimethylated adenine (DMA) was adapted from the m^6^A library (46). The 20 CYANA-minimized structures with the lowest target functions were refined with the leaprc.ff14SB force field using the sander module of AMBER14 (47). The force field for m^6^_2_A applied here was leaprc.modrna08 from the amber library, obtained from Aduri *et al.* (48) (http://oznoe3.chem.wayne.edu). PyMOL (http://www.pymol.org) was used for structural analyses.

## RESULTS

### hsTFB1M shares a common fold with rRNA adenine methyltransferases

**Figure 1.**
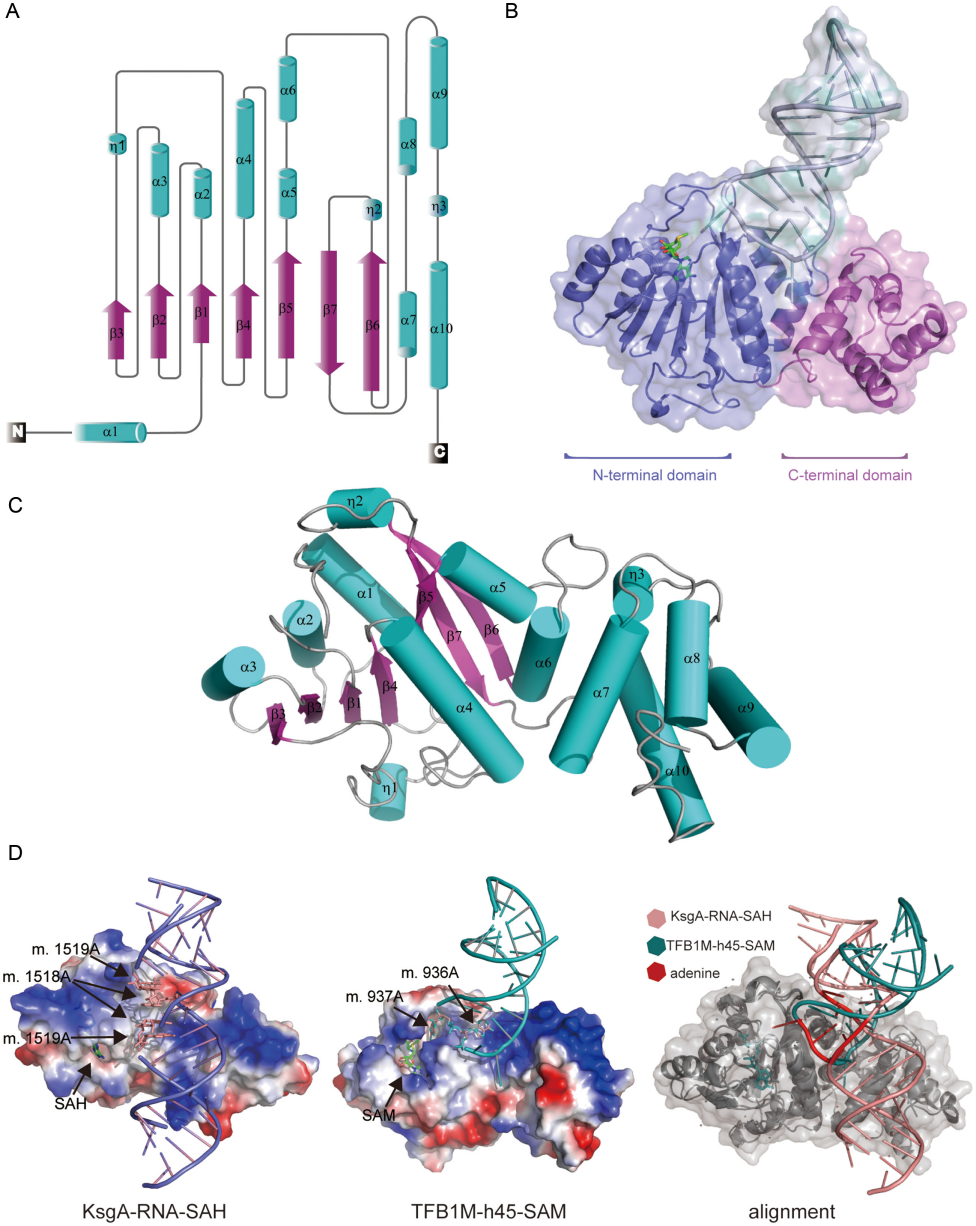
Structural analysis of hsTFB1M in the hsTFB1M–h45–SAM complex. (**A**) Topological graph of hsTFB1M lacking signal peptide. β-sheets are colored in magenta, and α-helices are colored in cyan. (**B**) The overall structure of the hsTFB1M–h45–SAM ternary complex. HsTFB1M is a member of the *N^6^*-methyltransferase family and contains an N-terminal domain (blue) and a C-terminal domain (pink). The S-adenosylmethionine (SAM) molecule is colored in green and h45 is colored in gray. (**C**) Schematic representation of hsTFB1M. Colors are as in (A). (**D**) Left diagram shows the structure of the KsgA–RNA–SAH complex of *A. aeolicus* (slate for RNA; PDB ID: 3ftf). Two RNAs are shaped into a duplex through complementary pairing, in which the ‘GGAA’ loop forms four mismatched G–A base pairs. Middle diagram shows our structure of the TFB1M–RNA–SAM complex from *H. sapiens* (deep teal for RNA; PDB: 6AAX). A single RNA folds into a stem loop, and m. 937A inserts into the pocket. The important elements are marked with black arrowheads. The alignment of these two structures is shown in the right diagram, and the red color shows the adenines that will be modified.

To study the mechanism of m^6^_2_A modification, we cloned, expressed, and purified hsTFB1M, transcriptionally purified the 28-nucleotide unmodified h45 RNA *in vitro*, and crystallized and determined the structures of a binary (hsTFB1M–h45) and ternary complex (hsTFB1M–h45–SAM) at a resolution 3.0 Å. Comparison of these two complexes revealed identical fold conformation of hsTFB1M and h45 appearing at an approximate 10° rotation only in the stem, possibly formed by base stacking (Supplementary Figure S1D). Therefore, we mainly used the ternary complex (hsTFB1M–h45–SAM) for subsequent description. Similar to all rRNA adenine methyltransferases, hsTFB1M showed a two-domain structure in the ternary complex (Figure 1B). The N-terminal domain (residues 28–236; residues 33–236 could be seen in the structure) formed a canonical Rossman-like methyltransferase fold with a central seven-stranded β-sheet flanked by three α-helices on each side. The C-terminal domain (residues 237–324) contained four α-helices (α7–α10) that likely formed a scaffold for other functional proteins (Figure 1A–C). The active center, located in the N-terminal domain, was equally defined by two α-helices (α1–α2) and a loop region connecting two β-sheets (β6–β7). As a typical SAM-dependent methyltransferase, hsTFB1M exhibited a conserved negatively-charged SAM-binding pocket surrounded by Glu85, Lys86, Asp111, Val112, Asn141, Leu142 and Val146 (Supplementary Figure S1C), whose primary role was establishing hydrogen bonding and hydrophobic interactions. Two hydroxyl groups in the ribose moiety of SAM formed bidentate hydrogen bonds with the carboxyl group of Glu85. Adenosine N^6^ formed the same interactions with Asp111. Adjacent hydrophobic residues (Val112, Leu142 and Val146) provided the bulk of the hydrophobic interactions with the SAM substrate. Lys86 may establish van der Waals interactions to stabilize the adenine base. Asn141 mainly interacted with SAM via its main chain and also helped to form the pocket for m. 937A. All of these residues (either their sequences or their structures) were conserved between prokaryotes and eukaryotes (Supplementary Figures S2 and S3A). Alignments of TFB1M structures from *H. sapiens* (PDB ID: 6AAX), *Mus musculus* (PDB ID: 4gc9), *E. coli* (PDB ID: 1qyr) and *Aquifex aeolicus* (PDB ID: 3ftf) showed near-complete conservation of residues near the SAM/S-adenosylhistidine (SAH) binding pocket (Supplementary Figure S3B). Interestingly, the molecules bound in this pocket included both SAM and SAH, with SAH observed in structures of most prokaryotic enzymes and SAM in those of eukaryotes. We hypothesized this substrate difference could be related to functional complex formation in eukaryotes, which we have clarified below. Moreover, differences between the structures of TFB1M and KsgA were observed in loop I and loop II, located between β3 and β4 and α4 and β5, respectively (Supplementary Figure S3A). Both loops were longer in eukaryotes. Loop I contained a short helix in *H. sapiens* and *M. musculus*, but it was just a long loop in prokaryotes, indicating that evolutionary differences existed among these homologous proteins.

### Folding of h45 in the hsTFB1M–h45–SAM complex is different from the KsgA–RNA–SAH complex

**Figure 2.**
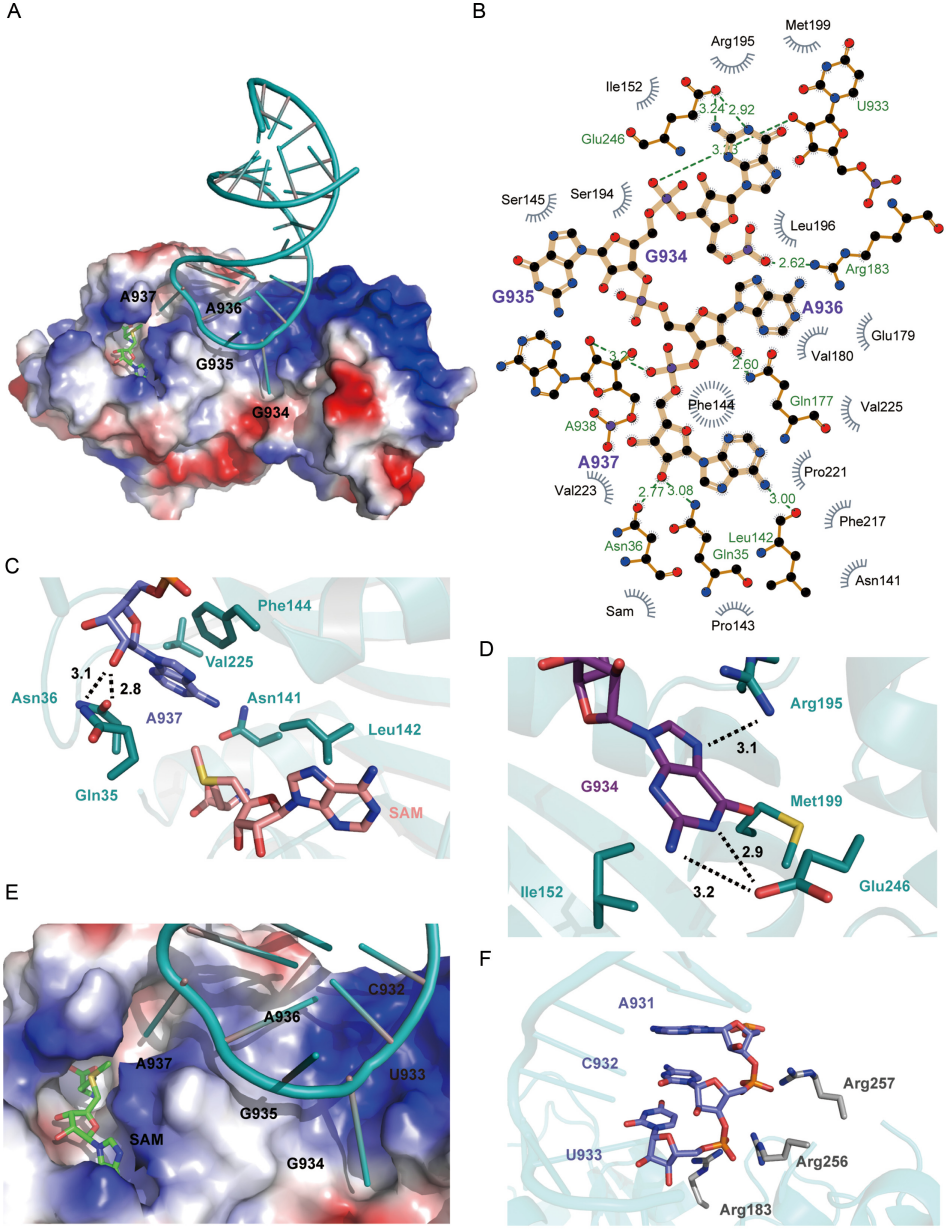
Detailed characterization of the interaction between hsTFB1M and h45. (**A**) Electrostatic surface potential maps of hsTFB1M in complex with h45 (deep teal) and SAM (green). (**B**) Interaction plot between TFB1M and ‘GGAA’ tetraloop of h45 (plot produced using LigPlot^+^ v. 1.4). (**C**) Sticks model for the pocket containing m. 937A (blue). The interacting residues are colored in deep teal and the SAM molecule is colored in deep salmon. Black dashed lines show hydrogen bonds. (**D**) Interactions between m. 934G (deep purple) and a tiny pocket on hsTFB1M. (**E**) Schematic diagram for the interaction between the h45 GGAA tetraloop and hsTFB1M. (**F**) Electrostatic interactions between the phosphate backbone of m. 931A, m. 932C and m. 933U (slave) and hsTFB1M residues Arg183, Arg256 and Arg257 (gray), respectively.

The structure of the ternary complex (hsTFB1M–h45–SAM) clearly showed how the substrate RNA, h45, interacts with hsTFB1M. In the structure, h45 folded into a hairpin structure capped with a typical four-nucleotide ‘GGAA’ tetraloop and was suspended from hsTFB1M, stretching across its N-terminal and C-terminal domains. The interaction between h45 and hsTFB1M mainly depended on electrostatic interactions mediated by the ‘GGAA’ tetraloop and the h45 phosphate backbone (Figure 2A and E). Electrostatic potential surface maps showed a positively-charged region located in the C-terminus of hsTFB1M, with interactions formed between Arg183, Arg256, and Arg257 and the phosphate backbone of h45 (Figure 2F). The effect of these interactions was to stabilize the RNA for specific bases to be modified by the enzyme active site. The combined form of the RNA in our crystal structure was quite different from the complex studied by Tu *et al.* (49) in *A. aeolicus*, wherein two identical RNA molecules formed a duplex with four mismatched G–A pairs in the middle (Figure 1D). In that complex, the candidate adenines for dimethylation were in a base-paired state and could not enter the enzyme active center to be modified. Conversely, in our structure, h45 formed a stem loop and presented one adenine into the hsTFB1M active pocket. Based on the location of h45 in the 12S rRNA, we assume that the folding mode of h45 in the hsTFB1M–h45–SAM complex more accurately reflects the true enzymatic state.

### m. 937A is preferentially recognized by hsTFB1M in the initial state

Both clinical and experimental evidence showed that m. 936A and m. 937A could be dimethylated by TFB1M (28,50). Four methyl groups would eventually replace the *N^6^* hydrogen atoms of two adenines, but the molecular events underlying this process were unknown. To explain this process, we used structural analysis of the initial state. Interestingly, the structure of the hsTFB1M–h45–SAM complex showed that unmethylated m. 937A was inserted into the enzyme active-site pocket in the initial state. Moreover, the active-site pocket, formed by the highly-conserved residues Gln35, Asn36, Asn141, Phe144 and Val225, could only accommodate one nucleotide of h45 (Figure 2C). As is the general case for m^6^A recognition, π–π stacking interactions between the adenine base and the aromatic cage were also facilitated in this pocket, mainly through m. 937A and Phe144. The hydroxyl group of the adenine ribose moiety established hydrogen bonds with Gln35 and Asn36. Asn141, also involved in the formation of the SAM-binding pocket, potentially mediated the interaction between adenine and SAM. In the structure of the hsTFB1M–h45–SAM ternary complex, SAM lay in a conserved acidic pocket immediately adjacent to the m.937A pocket in order to present the SAM methyl group to one of the two *N^6^*H atoms in m.937A (Figure 2C). Another modified target m. 936A was lying on the opposite side, preparing for subsequent entry into the active-site pocket.

### m. 934G is anchored within a tiny pocket in hsTFB1M

The first guanine in the ‘GGAA’ tetraloop m. 934G also stretched into a tiny pocket surrounded by Ile152, Arg195, Met199 and Glu246 (Figure 2D). Glu246 and Arg195 formed hydrogen bonds primarily with the base of m. 934G. Ile152 and Met199 potentially formed hydrophobic and van der Waals interactions, respectively. Based on the distance between the first guanine and the last adenine in the tetraloop, m.934G was a possible recognition site for the initial anchoring of h45 to hsTFB1M, and this was likely to cause m.937A to stretch into the active pocket.

### Binding between h45 and hsTFB1M primarily depends on electrostatic interactions

**Figure 3.**
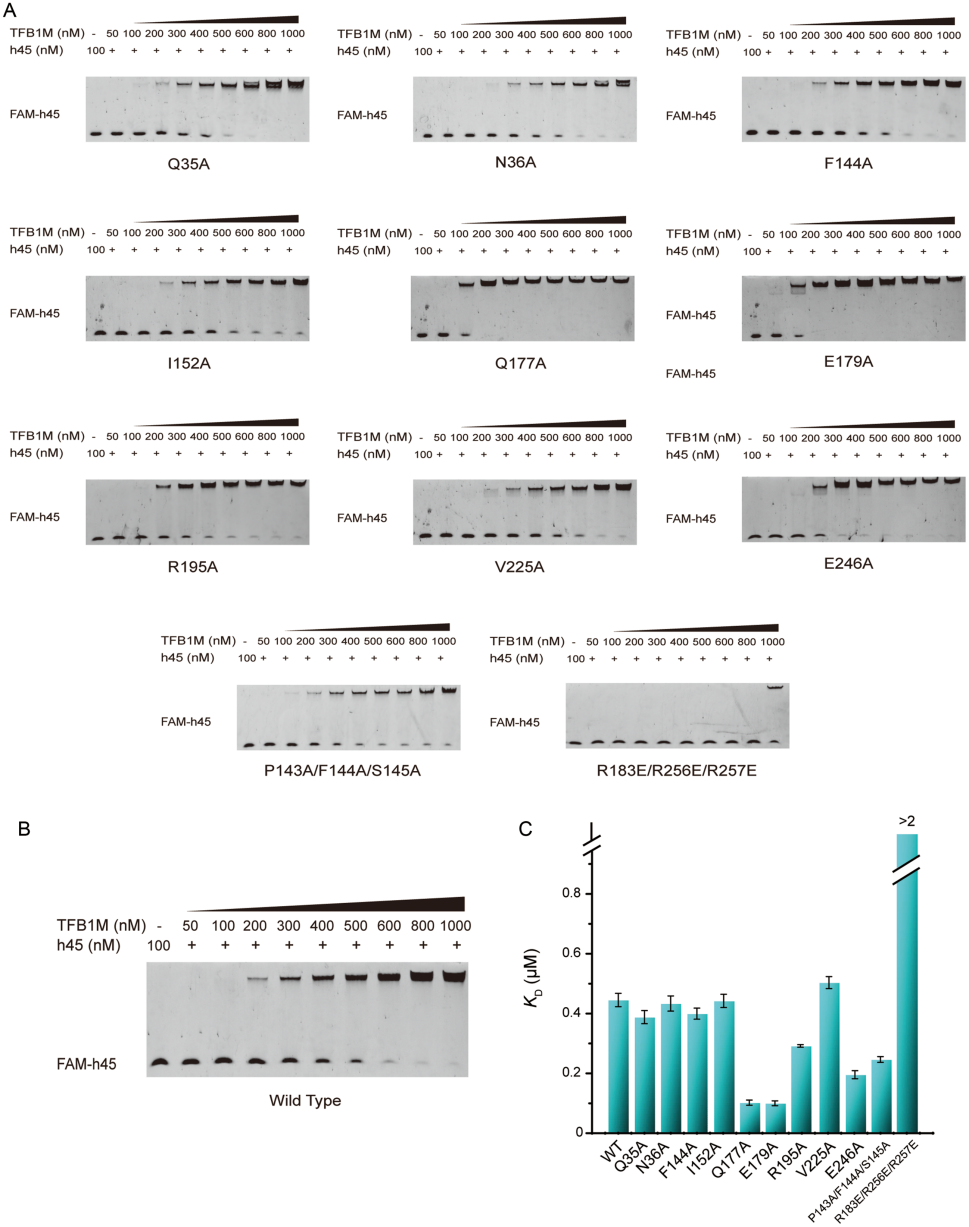
Electrophoresis mobility shift assay (EMSA) analyzing the affinity between unmethylated h45 and hsTFB1M (wild-type or mutants). (**A**) Mutants of key positions in hsTFB1M. According to the crystal structure, hsTFB1M mutants bearing Q35A, N36A, V225A, and F144A substitutions were predicted to abolish the hydrogen bonding or hydrophobic interactions with m. 937A of h45, with the F144A substitution destroying critical π–π stacking interactions in the m. 937A pocket. I152A, R195A, and E246A represent substitutions of residues close to m. 934G, and Q177A and E179A were residues neighboring m. 936A. To further assess whether binding depended on multiple sites, we also mutated significant pocket and binding motifs, including P143A/F144A/S145A and R183E/R256E/R257E. (**B**) EMSA of h45 and wild-type hsTFB1M. (**C**) Schematic of apparent *K*_D_ values for all the mutants. Apparent *K*_D_ values were fitted with Origin 8.5 software. Only mutants R183E/R256E/R257E abolished the interaction between h45 and TFB1M and their apparent *K*_D_ values were far greater than 2 μM.

The crystal structure showed that the interactions between h45 and hsTFB1M relied on the ‘GGAA’ tetraloop, particularly m. 937A and m. 934G. As shown in Figure 2E, m. 937A inserted into the active-site pocket in the hsTFB1M N-terminal domain, and m. 934G entered another pocket in the C-terminal domain. However, according to the electron density map for h45, the remaining two nucleotides, m. 935G and m. 936A, were relatively dynamic. The base of m. 935G could not be clearly identified in the electron cloud and was lying in the opposite direction of the binary and ternary complex (Supplementary Figure S1B), implying this guanine was structurally dynamic; this finding was exactly in line with the subsequently-determined NMR structure. Moreover, in the ternary complex, m. 936A was lying on hsTFB1M awaiting modification, showing meager distinction in the extension direction of its base, suggesting that it was relatively flexible and did not interact significantly with hsTFB1M. Thus, hydrogen bonding interactions in the m. 937A- and m. 934G-binding pockets, along with electrostatic interactions, were supposedly the primary determinants of binding between enzyme and nucleotide. Surprisingly, EMSAs revealed that alanine substitution of critical residues in the hsTFB1M m.937A-binding pocket, including Gln35, Asn36, Phe144 and Val225, did not significantly affect the binding affinity of hsTFB1M for h45 (Figure 3A and B). We also substituted several key hsTFB1M residues involved in hydrogen bonding or hydrophobic interactions with the ‘GGAA’ tetraloop with alanine (Figure 2B) and found that loss of Ile152, Arg195, and Glu246 close to m. 934G did not impair the binding affinity. Instead, alanine substitution of Gln177 and Glu179 near m. 936A improved the affinity of the interaction to some degree, potentially due to changes in local protein charge (Figure 3A and C). To further define the interaction mode between hsTFB1M and h45, we simultaneously mutated multiple sites on the binding surface by substitution of aromatic and hydrophilic residues with alanine (P143A/F144A/S145A) and positively-charged residues with glutamic acid (R183E/R256E/R257E). Surprisingly, only substitution of positively-charged residues (R183E/R256E/R257E) significantly impaired the binding affinity of hsTFB1M for h45 (Figure 3A and C). Therefore, we assumed that binding between hsTFB1M and h45 primarily depended on electrostatic interactions.

### Mutations of the enzyme active site or the binding interface impede the modification of h45

**Figure 4.**
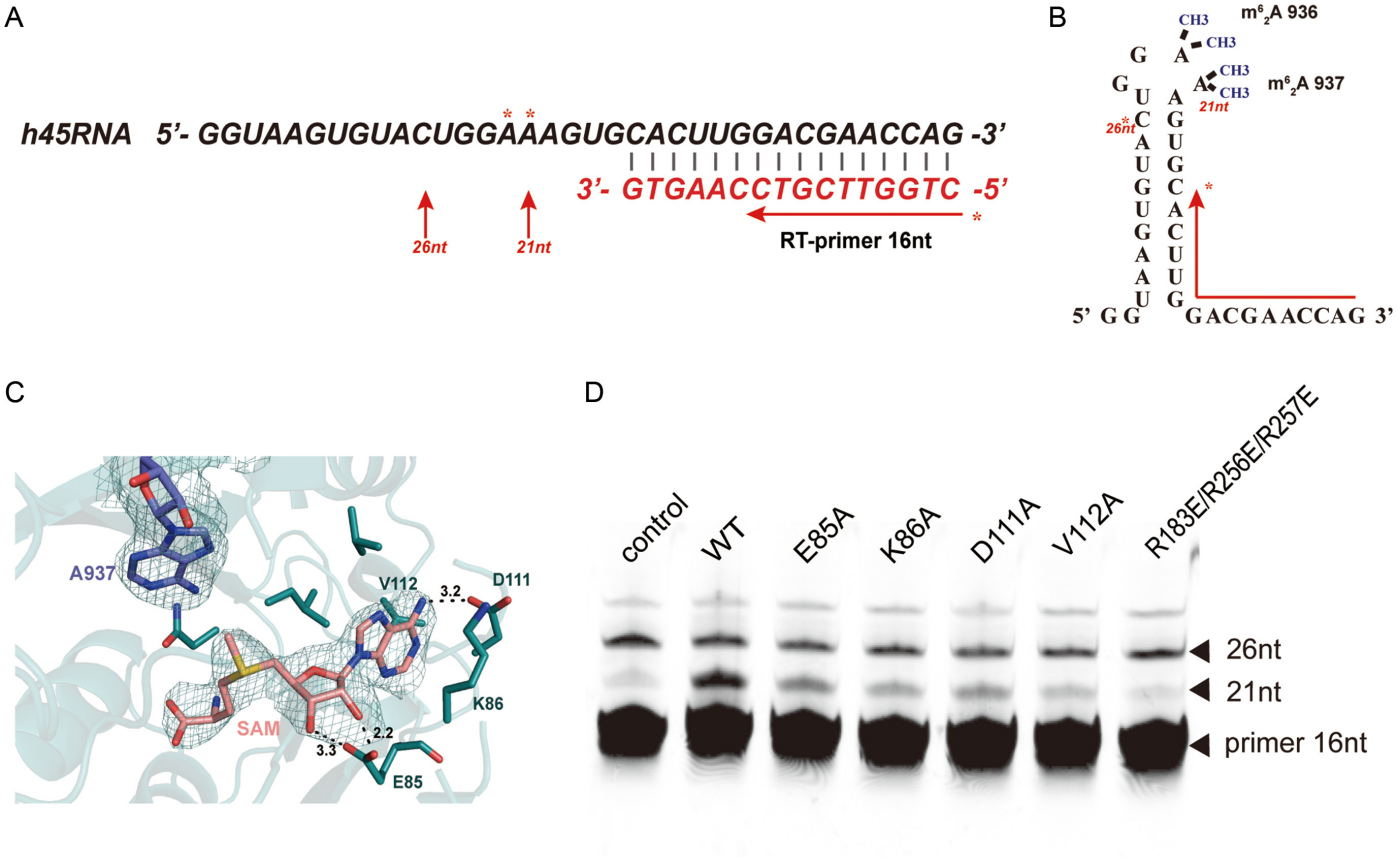
Primer extension analysis of human mitochondrial 12S rRNA. (**A**) Schematic showing sequence of human 12S helix 45 (h45; black letters) and the RT-primer (red letters) for the primer extension assay. Two dimethylated adenines are marked with asterisks. The red arrows point out the stop locations at modification and read-through sites. (**B**) The secondary structure of 12S rRNA h45 in accordance with (A). The two dimethylated adenines are marked with methyl groups and recorded as m. 936A and m. 937A. The primer (16 nt) used in this experiment is indicated by bent arrows, and was complementary to the RNA sequence. The asterisk beside the arrow shows the last G in the primer and that next to C shows the 26-nt read-through product. A 21-nt truncation product will be identified if m. 937A is modified. (**C**) The view of the pocket for m. 937A (blue) and SAM (pink) is the same as in Figure S1C. The key residues near the enzyme active site were mutated to detect enzyme activity in (D). (**D**) Primer extension was performed to assess the modification state of h45. TFB1M complex was extracted by GST-TFB1M pull-down from PLC cells and then used to complete the catalytic reaction *in vitro*. The control lane represents a reaction system without the TFB1M complex.

We used primer extension assays (28) to evaluate the modification of h45 by TFB1M complex obtained from hepatoma carcinoma cells. The identification of gel banding patterns showed that extension stopped mainly at 21-nt length, which is at the site of modified m. 937A (Figure 4A and B). The band intensity for mutants of the enzyme active site (E85A, K86A, D111A and V112A), as well as substitutions preventing enzyme-RNA binding (R183E/R256E/R257E), was significantly weaker than that for the WT enzyme (Figure 4C and D). Thus, h45 modification was impaired by TFB1M substitutions that inhibited enzyme activity or blocked binding between enzyme and substrate.

### TFB1M deficiency impairs ATP production and suppresses mitochondria protein translation

**Figure 5.**
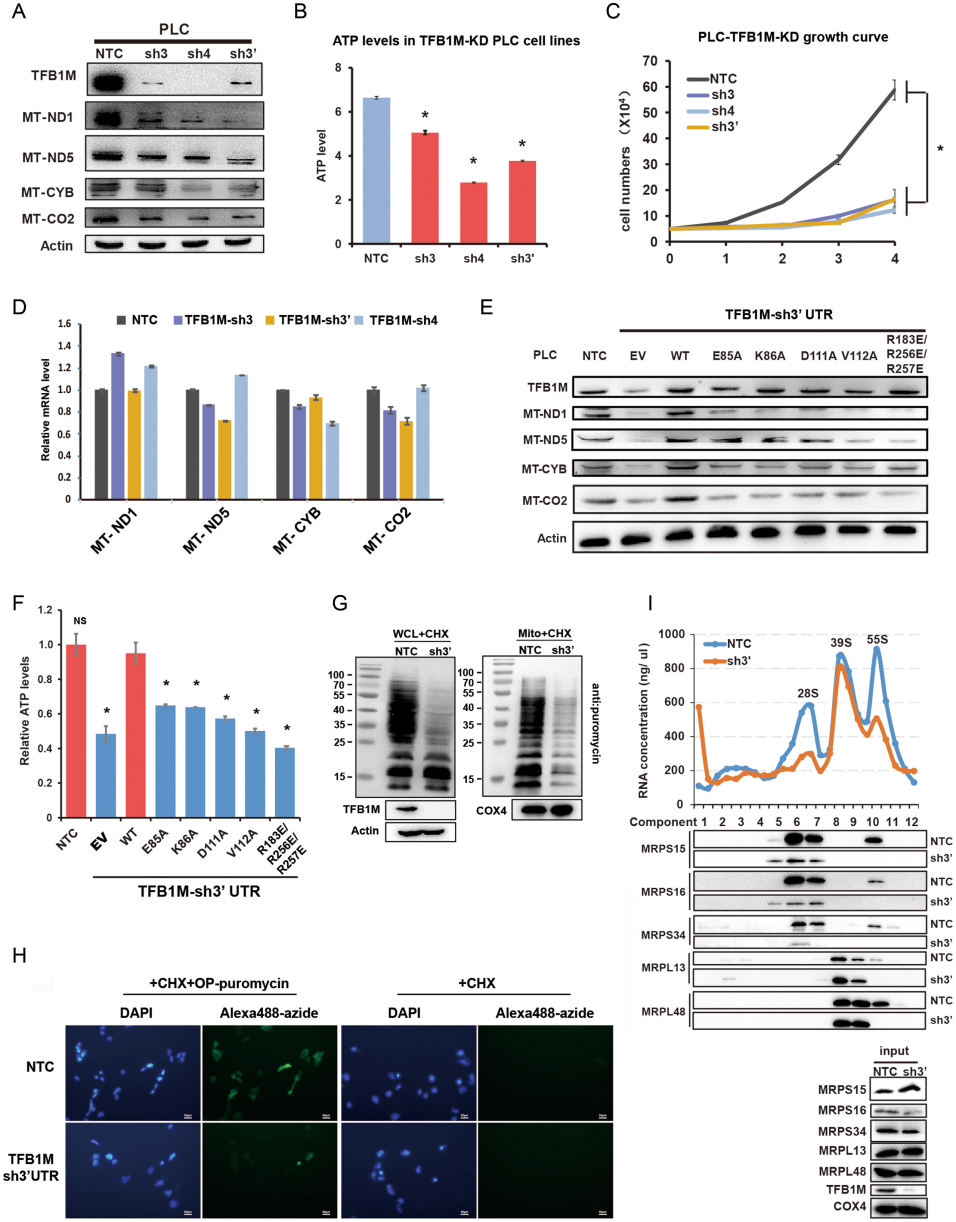
Analysis of mitochondrial function after hsTFB1M knockdown in hepatoma carcinoma (PLC) cells. (**A**) Protein levels of hsTFB1M and mitochondrial gene-encoded proteins in OXPHOS complexes, including complex I NADH-ubiquinone oxidoreductase chain 1 (MT–ND1), MT-ND5, complex III cytochrome (MT-CYB), complex IV cytochrome c oxidase 1 (MT-CO1), and MT-CO2, were determined in PLC cells. Two shRNAs (sh3 and sh4) targeting the coding sequence of TFB1M, and one shRNA (sh3′) targeting the 3′-UTR of TFB1M were used to knockdown TFB1M. β-actin served as loading control. NTC: non-targeting control. (**B**) The cellular ATP levels of PLC cells expressing shTFB1Ms were assessed using an ATP assay kit as described in Materials and Methods. (**C**) The growth curves of PLC cells expressing shTFB1Ms. Knockdown efficiency in PLC cells was verified by western blotting (shown as the TFB1M lane in (A)). (**D**) Abundance of mRNA transcripts encoding mitochondrial polypeptides including MT-ND1, MT-ND5, MT-CYB and MT-CO2 were determined by quantitative real-time PCR in PLC cells wherein TFB1M was knocked down. The mRNA levels were normalized to 18S. (E, F) PLC cells in which endogenous TFB1M was silenced using a shRNA targeting the 3′UTR (sh3′UTR) were further infected with viruses expressing TFB1M (wild-type, active-site mutants E85A, K86A, D111A, or the combined mutants of the interface between TFB1M and h45, R183E/R256E/R257E) followed by analysis of the cellular ATP (**F**) and mitochondrial encoded protein analysis by western blotting (**E**). (G, H) PLC cells expressing NTC or shTFB1M were treated with CHX firstly and then with puromycin. Whole cell lysates or purified mitochondrial lysate were subjected to Western blot with anti-puromycin antibody as a measure of actively translated polypeptide chains. TFB1M blot confirmed the knockdown effect of shTFB1M. COX4 and Actin served as loading controls (**G**). The signal of Alexa 488 azide showed the abundance of OP-puromycin labeled proteins and presented the rate of synthesis of mtDNA-encoded proteins (**H**). The cells without OP-puromycin treatment serve as negative control and all the signal of Alexa 488 azide were exposed for the same time(1s) and under the same condition. (**I**) The same amount of mitochondria from NTC- or shTFB1M-expressing PLC cells treated with CHP and TAP were loaded onto the sucrose density gradient. After centrifugation, fractions of gradients were subjected to western blot analysis and RNA concentration measurement. TFB1M blot confirmed the knockdown efficiency of shTFB1M. COX4 served as loading controls. *P < 0.05 as compared to the NTC control group.

To further study the biological function of m^6^_2_A-h45 modification in mitochondria, we explored the impact of TFB1M knockdown on transcription and translation of genes and proteins that play important roles in electron transport and OXPHOS. The mtDNA genome encodes 13 indispensable proteins that form parts of complexes I, III, IV and V and perform cellular respiration and ATP synthesis. Therefore, levels of several mitochondrial genes and proteins were analyzed in stable PLC cell lines bearing TFB1M knockdown. Western blotting with antibodies against TFB1M, complex I NADH-ubiquinone oxidoreductase chain 1 (MT-ND1), complex I NADH-ubiquinone oxidoreductase chain 5 (MT-ND5), complex III cytochrome b (MT-CYB), and complex IV cytochrome *c* oxidase 2 (MT-CO2) showed that their expression decreased when TFB1M was knocked down in PLC cells (Figure 5A). Thus, TFB1M positively regulated the expression of mitochondria gene-encoded proteins in OXPHOS. However, quantitative real-time PCR indicated that the corresponding mRNA levels of these mitochondrial genes remained stable irrespective of TFB1M knockdown in PLC cells (Figure 5D). This result implied that the suppression of TFB1M impaired translation, rather than transcription, of mitochondrial genes involved in OXPHOS. Because the efficiency of OXPHOS is related to ATP production *in vivo*, we assessed the cellular ATP production in PLC cells and found that ATP levels were significantly decreased when TFB1M was silenced using shRNAs (Figure 5B). Moreover, the proliferation of PLC cells with TFB1M knockdown was also markedly suppressed (Figure 5C). These results documented that TFB1M regulated OXPHOS and ATP production *in vivo*.

To confirm the effect of m^6^_2_A-h45 modification defects *in vivo*, we knocked down endogenous TFB1M using sh3′ UTR (designed to target the 3′ untranslated region of TFB1M) RNA in PLC cells wherein WT or mutant TFB1M (E85A, K86A, D111A, V112A and R183E/R256E/R257E) were overexpressed. Western blotting experiments demonstrated that forced expression of WT TFB1M elevated shTFB1M-decreased mitochondrial gene-encoded proteins, whereas TFB1M mutants exhibited no rescue effect (Figure 5E). Consistently, cellular ATP analysis revealed that the forced expression of WT TFB1M rescued shTFB1M-suppressed cellular ATP production, whereas TFB1M mutants showed no rescue effect (Figure 5F). In addition, immunofluorescence analysis confirmed that as was the case for WT TFB1M, all TFB1M mutants were still localized to mitochondria (Suppleemntory Figure S3E). More importantly, we determined the rate of synthesis of mtDNA-encoded proteins in NTC and TFB1M knockdown PLC cells using the SUnSET assay and OP-Puro labeling, which evaluate the incorporation of puromycin or OP-puromycin in nascent proteins (51–53). In SUnSET assay and OP-puromycin labeling assay, we treated cells with cycloheximide (CHX) which blocks cytoplasmic ribosome-mediated protein translation, so the puromycylation reaction only placed puromycin tags or OP-puromycin tags into mt-DNA encoded nascent peptides undergoing active translation and produces prematurely terminated peptides. Therefore, all the puromycin bands detected by western blot and green fluorescence from OP-puromycin labeling represented the newly synthesized polypeptides randomly incorporated with puromycin in mitochondria. Our data showed that knockdown of TFB1M markedly decreased the puromycin signal compared to non-targeting control (NTC) group in both the SUnSET (Figure 5G) and OP-Puro labeling (Figure 5H) assays, documenting that suppression of TFB1M inhibited the rate of mitochondrial protein synthesis.

To study the effect of TFB1M on the mitochondrial ribosomes, we isolated and lysed the mitochondria from PLC cells expressing NTC or shTFB1M and separated the lysate on sucrose gradients. After centrifugation, fractions of gradients were subjected to western blot analysis and RNA concentration measurement. As a result, RNA concentration measurement showed that suppression of TFB1M reduced the mitochondrial 28S small subunit (mt-SSU) but not the mitochondrial 39S large subunit (mt-LSU). Consistently, western blot analysis revealed that knockdown of TFB1M markedly suppressed the protein levels of mt-SSU (MRPS15, MRPS16, MRPS34), but not that of mt-LSU (MRPL13, MRPL48), supporting the conclusion that knockdown of TFB1M suppressed the assembly of mitochondrial small 28S subunit, but not that of mitochondrial large 39S subunit. Moreover, we also observed that suppression of the mitochondrial small 28S subunit assembly led to decreased mitochondrial monosomes (Figure 5I). Thus, taken together, these data suggest that the methylation enzymatic activity of TFB1M, as well as its interaction with RNA, is critical for its ability to regulate mitochondrial protein translation and cellular ATP production.

### Solution structure of unmodified RNA h45 forms a standard A-form helix

**Figure 6.**
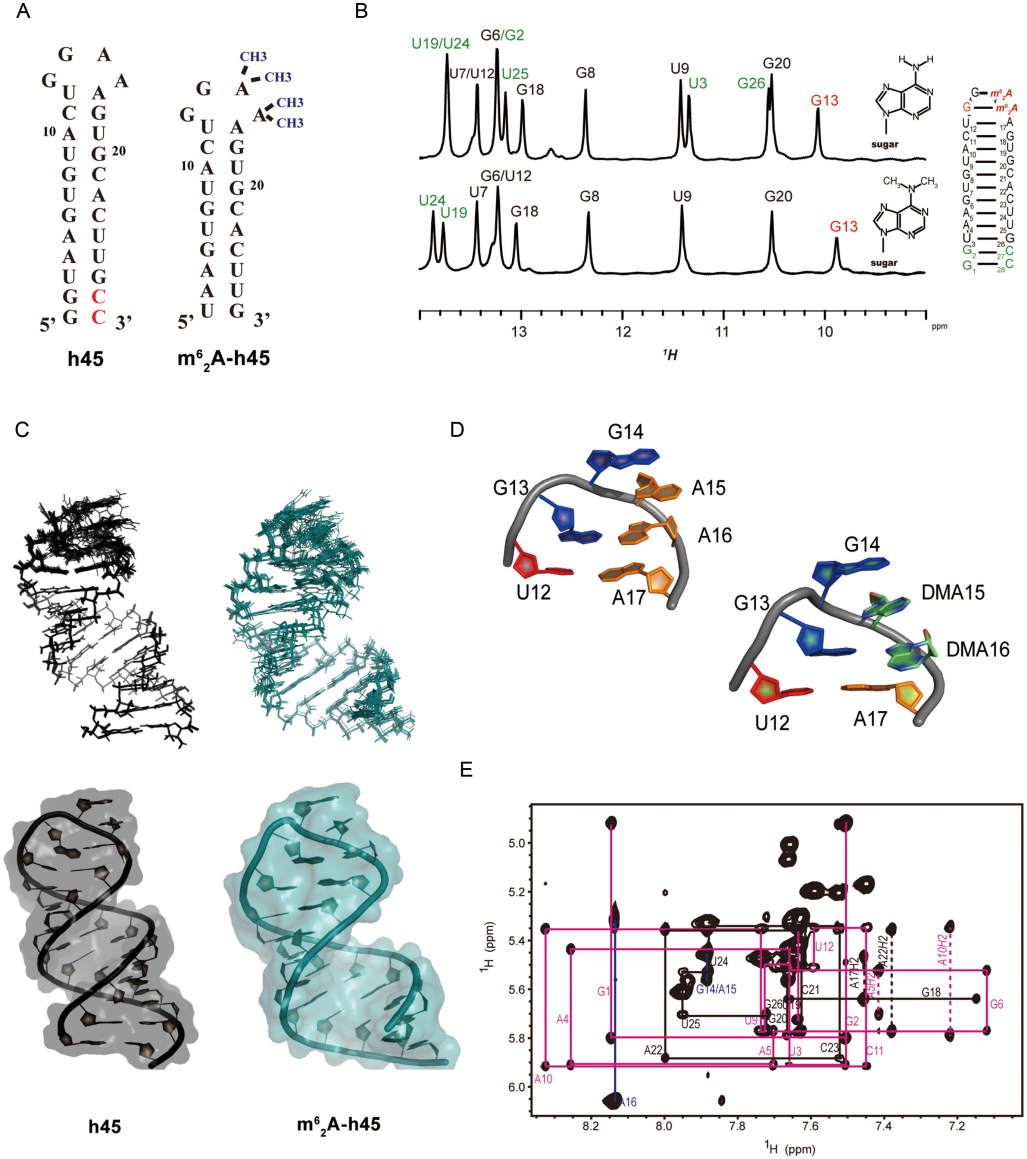
Solution structures of 12S rRNA helix 45. (**A**) Secondary structures of h45 and m^6^_2_A-h45, renumbered as 1–28 and 3–24, respectively. The m^6^_2_A-h45 RNA contains four methyl groups in the ‘GGAA’ tetraloop. (**B**) 1D spectra for h45 and m^6^_2_A-h45. Comparison of imino peaks showed the same peak positions for most of base pairs in the stem region except the terminal two base pairs. The first nucleotide in the loop, G13, had a slightly different chemical shift and weaker intensity after dimethylation. (**C**) NMR solution structures for the superposition of the 20 lowest energy conformations (upper) and the single lowest energy structure (lower). The colors for h45 and m^6^_2_A-h45 are black and deep teal, respectively. (**D**) Close-up view of the ‘GGAA’ tetraloop of h45 presented as a ribbon-and-stick model. Nucleotides are colored red (uracil in the adjacent pair), blue (guanine in loop), green (adenine in loop), and orange (adenine in the adjacent pair). (**E**) Portion of 2D ^1^H-1H NOESY in D_2_O showing the ‘walk’ pattern for the h45 stem region. Sequential NOE connectivity of cross-peaks between aromatic H6/H8 and ribose H1′ are indicated by pink lines for the 5′ half (G1 to U12), by black lines for the 3′ half (A17 to C28), and by blue lines for the ‘GGAA’ tetraloop.

According to the crystal structure, the ‘GGAA’ tetraloop flipped out to interact with hsTFB1M. To clarify whether h45 underwent conformational changes pre- and post-dimethylation, we determined the solution structures of h45 with (m^6^_2_A-h45) and without (h45) modification using NMR spectroscopy. The sequence of h45 used in this structure corresponded to nucleotides 922–949 of the mitochondrial 12S rRNA of *H. sapiens*, which is capped by the ‘GGAA’ tetraloop on the top of its stem and is terminated at two cytosines at positions 948 and 949 for efficient transcription. For convenience, the nucleotides were renumbered from 1 to 28 for unmodified h45 and from 3 to 26 for dimethylated h45 (Figure 6A). Assignments for the ^1^H, protonated-^13^C and -^15^N spectra in unmodified h45 were accomplished by reference to homonuclear and heteronuclear 2D or 3D NMR spectra of unlabeled along with uniformly ^13^C- and ^15^N-labeled samples in H_2_O and D_2_O.

Resonances of imino protons from the stem residues of unmodified h45 were observed in the 2D NOESY spectrum in H_2_O starting from two G•U wobble base pairs (U3•G26 and U9•G20) at the upfield chemical shift of imino proton region and further confirmed using 2D HNN-COSY (Supplementary Figure S4A–C). Intriguingly, there was a medium diagonal imino peak for the first guanine (G13) of the tetraloop evident on the NOESY and 1D spectra, showing that a hydrogen bond connected the G13 with the last adenosine (A16) in the tetraloop; this result is in agreement with other ‘GNRA’ tetraloops (54,55). The aromatic H8/H6 to ribose H1′ ‘walk’ pattern was assigned from 2D NOESY in D_2_O, indicating that h45 forms an A-form helix in the stem region (Figure 6E). In addition, a sequential base H8/H6 to the ribose H1′ ‘walk’ pattern could also be observed in the tetraloop between U12 H1′ and G13 H8; thus, G13 may stack with the 3′ end of the left stem nucleotide U12. However, NOE between A15 H8 and G14 H1′ and between G14 H2′ and G14 H3′ suggested that stronger stacking interactions occurred between G14 and A15 than between A15 and A16, in which an interaction between A16 H8 and A15 H2′, H3′ can be observed (Supplementary Figure S4B). No stacking occurred between G13 and G14 due to absence of a sequential base H8/H6 in ribose H1′, H2′ and H3′, meaning that G14 is a turn corner for the tetraloop and is relatively dynamic. Additional dihedral angle restraints were obtained from the 2D DQF-COSY spectrum. All of the nucleotides in the stem, including the first G13 of the tetraloop, adopted *C3*′*-endo* A-form sugar pucker conformations. However, two intense cross-peaks of H1′–H2′, assigned as G14 and A15, apparently adopted the *C2*′*-endo* conformations.

### m^6^_2_A-h45 maintains a loop structure very similar to h45

Due to its commercial synthesis without protonated-^13^C and -^15^N, m^6^_2_A-h45 (24 nucleotides lacking among the G1–C28 and G2–C27 base pairs) was assigned only from a series of homonuclear 2D NMR spectra and by comparison with h45. Comparison of the 1D spectra of h45 and m^6^_2_A-h45 showed the same peak positions for most of the base pairs in the stem, but a slightly different chemical shift and weaker intensity for G13 after dimethylation (Figure 6B), indicating that the replacement of the methyl group in A16 did not cause the adenine to flip outside. The 2D NOESY spectra in D_2_O of m^6^_2_A-h45 still showed a clear ‘walk’ pattern in the base-pairing region, implying the A-form stem was also present in the m^6^_2_A-h45 structure (Supplementary Figure S4E). Interestingly, the imino peak for G13-m^6^_2_A16 was also maintained in the 2D-NOESY spectrum, like the 1D spectrum, but at a different chemical shift compared with G13–A16 (Supplementary Figure S4F). G14 appeared to be a dynamic nucleotide in h45 but still had base-stacking interactions with A15 and A16 to some degree. Moreover, m^6^_2_A15 still stacked on m^6^_2_A16 and together established hydrogen bonds with G13 using methyl groups rather than reaching out. This produced steric hindrance making G13 stretch toward the back side and caused congestion in the loop region (Figure 6C and D and Supplementary Figure S5). Only one diagonal peak could be observed on the 2D-NOESY spectrum in H_2_O for two methyl groups of m^6^_2_A15, yet two corresponding peaks were observed for m^6^_2_A16 (Supplementary Figure S4D). This finding potentially suggested that the methyl groups were exchanged relatively quickly in m^6^_2_A15 but slowly in m^6^_2_A16 because of the greater NOEs with the nucleotides in the stem region. G13–m^6^_2_A16 and the strong cross peak between the methyl hydrogen in m^6^_2_A16 and H2 in A17 provided the evidence for the base of m^6^_2_A16 not floating outside. In addition, the cross-peaks of H1′–H2′ for the four nucleotides in the tetraloop could not be seen in the 2D DQF-COSY spectrum for m^6^_2_A–h45 (data not shown), suggesting that all of the nucleotides in this loop may adopt *C3*′*-endo* sugar pucker conformations.

## DISCUSSION

### Dimethylation of h45 affects mitochondrial protein translation

**Figure 7.**
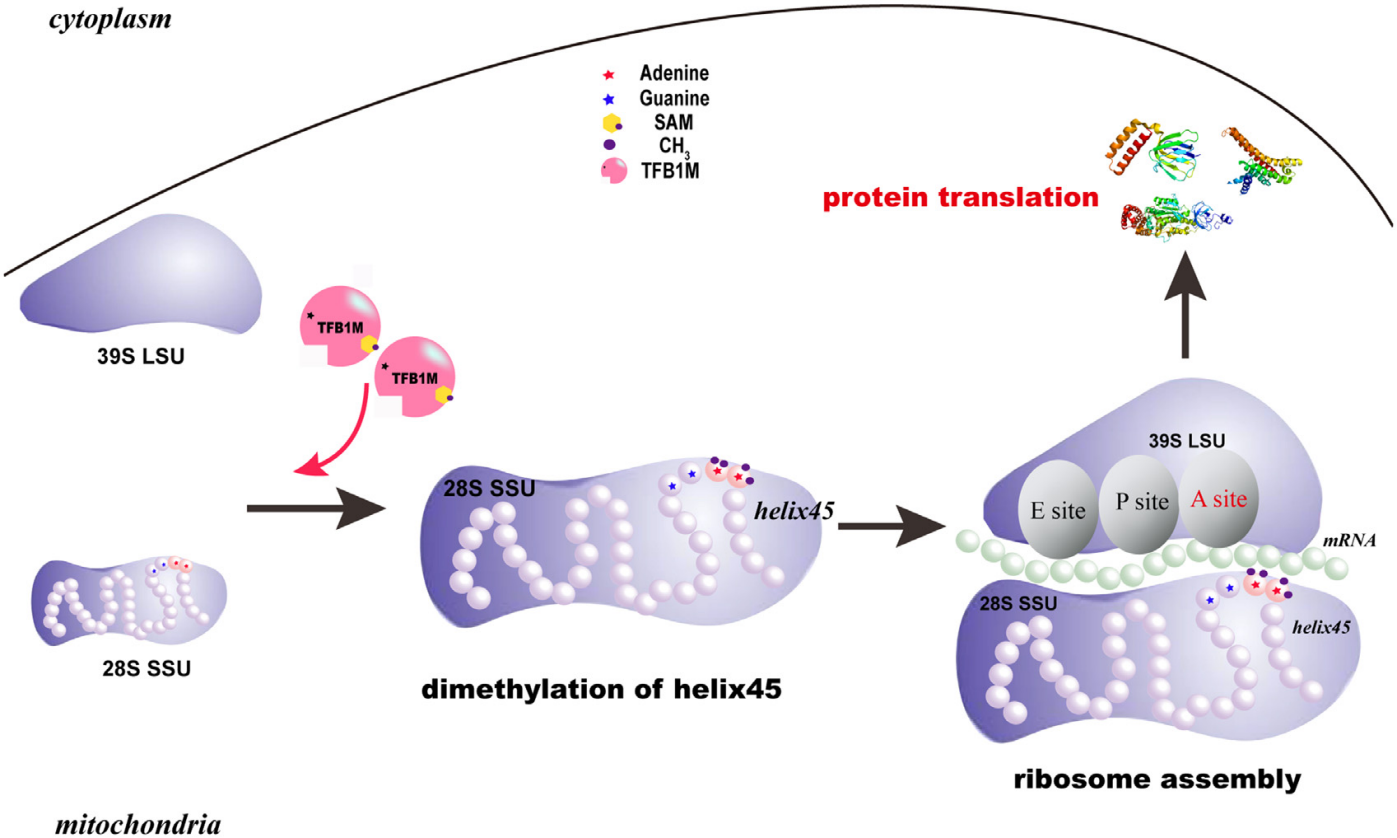
Model of m^6^_2_A dimethylation in the ribosome. Two adenines m. 936A and m. 937A of helix45 in 12S rRNA are folded into part of the scaffold of 28S small subunit and located in the surface of 28S SSU and 39S LSU; notably, the modification sites are close to the A-site. In mitochondria, when TFB1M with SAM binds helix45, the continuous adenines of the ‘GGAA’ loop are dimethylated, which promotes ribosome assembly and is followed by protein translation.

Dimethylation of the ‘GGAA’ tetraloop of h45 occurs in almost all organisms. It is believed that dimethylation site is located near A-site of ribosome; therefore, this m^6^_2_A post-transcriptional modification of h45 in 12S rRNA is likely a mark for ribosome assembly, further promoting efficient translation (9,12). In addition, its proximity to the interfaces of large and small subunits in ribosomes provides more possibility for influencing the function of ribosomes. Here our work offers further proof that the knockdown of TFB1M or mutants with lowered enzyme activity can abolish dimethylation of h45 in PLC cells (Figure 4C). This did not reduce the mRNA levels of essential genes related to mitochondrial respiration but did reduce the expression of the corresponding proteins. In TFB1M-knockdown PLC cells, rescue with mutant TFB1M also impaired the normal translation level of mitochondrial proteins. Lower ATP yields in TFB1M-deficient cells also provided evidence for mitochondrial dysfunction. Nevertheless, mutants of TFB1M did not influence the normal localization of mitochondria. Reduction of the enzyme activity of these mutants further demonstrated that dimethylation of h45 is a significant checkpoint for translation by ribosomes (Figure 7). Based on a report on ribosome-binding factor A (RBFA) (56), we consider that m^6^_2_A dimethylation of h45 in mitochondria is necessary for the completion of mitochondrial rRNA maturation and the regulation of the association of the small and large subunits, providing a new explanation for this mechanism. The m^6^_2_A post-transcriptional modification may be involved in a complex mechanism for the regulation of assembly of the two subunits of the ribosome.

### Differences in the enzyme active pocket of TFB1M and TFB2M may lead to functional preferences

The dual function proteins, TFB1M and TFB2M, participate in biogenesis as methyltransferases or transcription factors. Interestingly, how to distinguish between the two homologous proteins during the process of transcription and the reactions being catalyzed remains a topic worthy of study. The work of Metodiev has proven that TFB1M is not primarily a transcriptional factor but instead is an essential dimethyltransferase (9). Here, we provide potential structural evidence for the two functionally differentiated homologous proteins, TFB1M and TFB2M. In our structure of TFB1M and h45, when compared with other methyltransferases in prokaryotes, several key residues, including Glu, Lys, Asp, Val, Leu and Asn, could form a typical acid-active pocket accommodating SAM or SAH. However, TFB2M in the transcription initiation complex presents with similar folding but different enzyme active region from TFB1M. The key residues around the pocket are different except Glu and Asp, which form a significant bonding interaction with SAM (Supplementary Figure S3A and B). In addition, the enzyme active pocket in TFB1M exhibits a clear binding site for SAM, but the corresponding region in TFB2M is a highly positively-charged surface (Supplementary Figure S3C and D). We believe that this positive surface on TFB2M contributes to DNA binding and makes it function as an efficient transcription factor, whereas TFB1M reserves the SAM pocket for methyltransferase processing.

### Location priority of m. 937A in the initial state provides new insights into the mechanism of dimethylation

O’Sullivan *et al.* suspected that in 2014 m. 937A was dimethylated to m^6^_2_A by TFB1M before m.936A in *H. sapiens* based on primer extension assays in samples from deaf patients (50)_._ Our work provided further structural evidence for the priority of m. 937A recognition in the initial state: m. 937A lay in the center of the active pocket, close to the SAM pocket, to receive the first methyl group. However, anchoring of m. 934G in another tiny pocket may first be necessary, at sufficient distance for m. 937A to stretch into the reaction center (Figure 2C and D). Thus, the process of m^6^_2_A modification is possibly the same with m^6^A, during which the enzyme active center only accommodates one adenine and one SAM methyl group simultaneously. In the initial state, the recognition of unmodified RNA by dimethyltransferase may be different. The binding affinity analyses helped us to understand the TFB1M recognition motif. Electrostatic interactions between the phosphate backbones of h45 and positively-charged residues in hsTFB1M were critical for binding. Nevertheless, m. 934G plays a significant role. Inserting into a very tiny pocket, m. 934G is held by the pocket residues so as to stabilize binding of hsTFB1M and h45. In addition, m. 935G is relatively the most dynamic nucleotide of the tetraloop. Another modified target, m. 936A, lies on the opposite side, possibly preparing for subsequent entry into the active-site pocket. Therefore, m. 934G is likely to be a key recognition site for h45 by hsTFB1M and provides a suitable distance for m.937A to enter the active pocket for modification. Due to the limited distance between m. 934G and m. 937A, the conformation among the four nucleotides in the tetraloop may be changed obviously after m.937A methylation, a possibility that remains to be explored further. We tried to solve the complex crystal structures of hsTFB1M and h45 with different quantities of methyl groups, but could not obtain good diffraction data (data not shown). Further attempts to clarify the details of this modification mechanism structurally could be elements of future studies.

### HsTFB1M possibly functions as a complex to efficiently dimethylate h45 *in vivo*

*In vitro*, hsTFB1M alone showed scarcely any methyltransferase activity according to radioisotope labeling (data not shown). In addition, alignments of structures of this protein in prokaryotes and mammals suggest that active-site pockets bind a SAH molecule in the former but a SAM molecule in the latter organisms. This fact may imply that individual mammalian TFB1M molecules are insufficient to perform catalysis *in vitro*. The structural alignment of hsTFB1M and TFB2M (PDB ID: 6ERO) shows these two proteins have the same general architecture (Supplementary Figure S3C). In light of the transcription complex of mitochondria (30), we hypothesize that hsTFB1M possibly works in the form of a complex rather than alone. Therefore, we analyzed *in vitro* enzyme activity, employing complexes to catalyze the reaction. Cells of *E. coli* expressed TFB1M or mutants with GST-tag were collected and lysed. The supernatant was incubated with GST beads, and then the beads binding GST-tag proteins were incubated with NP40-lysed PLC cells to pull down the proteins interacting with TFB1M for catalyzation reaction. The results were subsequently detected by primer extension, and finally the length of modified RNA was assessed from the gel electrophoresis through the comparison with controls. Consequently, based on these analysis, we hypothesize that TFB1M employs as a complex in mitochondria to efficiently modify h45 *in vivo*.

Furthermore, Rozanska *et al.* (56) showed that human RBFA can bind helix 44 and h45 of 12S rRNA, possibly aiding h45 to present specific structures to TFB1M and promoting the maturation of the mitochondrial ribosome. During the biogenesis of mitochondrial small subunits, the chaperone Era like 12S mitochondrial rRNA (ERAL1) chaperones 12S rRNA to the small subunit, where RBFA binds it to facilitate m^6^_2_A dimethylation. Therefore, we assume that m^6^_2_A dimethylation is likely to involve multiple proteins *in vivo*. Nevertheless, we still do not have enough evidence on the interactions among the relevance of RBFA, ERAL1, TFB1M and other elements, which need to be studied further.

### ‘GGAA’ conformational changes between h45 and m^6^_2_A-h45 depend on the interaction with hsTFB1M

Our study showed that the solution structures of either h45 or m^6^_2_A-h45 had an A-form stem region capped by a standard ‘GGAA’ tetraloop, maintaining the G13–A16 hydrogen bonding pair (Figure 6C). G14 was the most dynamic nucleotide, which was in line with the conformation of m. 935G in the crystal structure. The NMR structure of m^6^_2_A-h45 comparing the 1D spectrum and the final conformation with h45 showed that peaks for most of the base pairs in the stem were nearly identical, but G13 had a diverse chemical shift. Therefore, the replacement of the methyl group in A16 did not cause the adenine to flip outside, and methyl groups only conferred steric hindrance to make the loop region more congested rather than causing major conformational changes. Conversely, in the crystal structure, the four nucleotides in the ‘GGAA’ tetraloop all flipped out in order to interact with hsTFB1M, either for anchoring or modification.

Clearly, the h45 or m^6^_2_A-h45 alone in solution state maintained a stable energy structure, but flipping out of the tetraloop may destabilize it in the absence of other interactions. We observed similar conformations of both molecules by NMR. However, when hsTFB1M participated in the process of modification, the candidate locations of ‘GGAA’ were required to enter the enzymatic center of the methyltransferase, a finding which may help us to understand the conformational changes in h45.

RNA post-transcriptional modifications have been proposed to affect transcription, translation, RNA editing, and other crucial metabolic processes in the cell. The roles of rRNA modifications in human mitochondrial ribosomes are not very well-established, and the effects of m^6^_2_A dimethylation still have not been clarified. In human mitochondria, this modification occurs at m. 936A and m. 937A of 12S rRNA h45, which is extremely conserved in sequence and structure. The relevant methyltransferases, KsgA in prokaryotes and TFB1M in mammals, belong to the class I-like adenine *N^6^*-methyltransferase family. Structures of *E. coli* KsgA (57) and *M. musculus* TFB1M (29) show the characteristic structures of methyltransferases. Subsequently, participation of duplex RNA in the structure of the KsgA–RNA–SAH complex from *A. aeolicus* shed new light on the binding of enzyme and substrate (49). The model for single-stranded RNA binding by TFB1M in *M. musculus* only supplied the hypothesis for this process (29). However, it is extremely important to clarify the binding mode between the TFB1M enzyme and its substrate to understand the mechanism of m^6^_2_A modification, and the previous reports were insufficient to understand the interaction between methyltransferases and their RNA substrates.

In the last decade, Mulder *et al.* have proved that the deficiency of TFB1M caused mitochondrial and β-cell dysfunctions, further inducing the risk of T2D, which was supposed to be due to the failure of dimethylation in 12S rRNA, and further destroying the mitochondrial ribosome assemble (33). In this study, we report the first ternary complex structure of human TFB1M, SAM and h45. In the structure, h45 forms a stem loop with the ‘GGAA’ tetraloop capped on the top of the stem but not as a duplex as in the KsgA–RNA–SAH structure. The latter structure showed two identical RNA molecules forming a duplex with four G–A mismatched base pairs in the middle such that none of the nucleotides could flip out to enter the active site of the enzyme (Figure 1D). Our complex structure provides a preliminary understanding of the mechanism of initiation of m^6^_2_A modification with m. 937A having priority to enter the enzyme active site in the initial state (Figure 2A, C, E). Moreover, m. 934G may play an important role in this process on account of being anchored within a tiny pocket at an appropriate distance for m. 937A insertion into the active site (Figure 2A, D, E). On comparing the solution structures of h45 with and without m^6^_2_A (Figure 6C), the conformational change of the ‘GGAA’ tetraloop, to a great extent, appeared to be affected by hsTFB1M binding. Subsequently, using hsTFB1M knockdown in hepatocellular carcinoma cells, we showed that the *in vivo* deficiency of m^6^_2_A modification impairs ATP production and reduces the expression of protein components of OXPHOS without affecting transcription of the corresponding genes (Figure 5D-F). Together, these data help us understand the m^6^_2_A modification both structurally and functionally. Therefore, we provided supplementary evidence that TFB1M deficiency led to the decline of m^6^_2_A modification in 12S rRNA, which resulted in the failure of ribosome assembly and translation in mitochondria, increasing the risk of T2D and other diseases. Given the inability of hsTFB1M to catalyze the m^6^_2_A modification *in vitro* and the possible complex of hsTFB1M *in vivo* that we hypothesize, further studies may focus on the detection of the process underlying this modification. Certainly, to understand the detailed mechanism of the m^6^_2_A modification, we need to continue our structural investigations with different methyl groups and find the association pathway of TFB1M and m^6^_2_A modification deficiency, which may be relevant to the study of mitochondrial diseases, along with T2D.

## DATA AVAILABILITY

Atomic coordinates of hsTFB1M–h45 and hsTFB1M–h45–SAM have been deposited in the Protein Data Bank under the accession code 6AJK and 6AAX, respectively. Coordinates for the 20 lowest energy structures of h45 and m^6^_2_A–h45 have been deposited in the Protein Data Bank under accession codes 6AAS and 6AAU, respectively. Chemical shifts have been deposited in the BioManResBank under accession codes 36202 and 27552, respectively.

## Supplementary Material

gkz505_Supplemental_File
