# Supplementary material for: Structural insights into dimethylation of 12S rRNA by TFB1M: indispensable role in translation of mitochondrial genes and mitochondrial function
Source: Nucleic Acids Res. 2019 Jun 28;47(14):7648–65. doi: 10.1093/nar/gkz505 (PMC6698656; doi:10.1093/nar/gkz505)
Supplement: gkz505_Supplemental_File [file gkz505_supplemental_file.pdf]

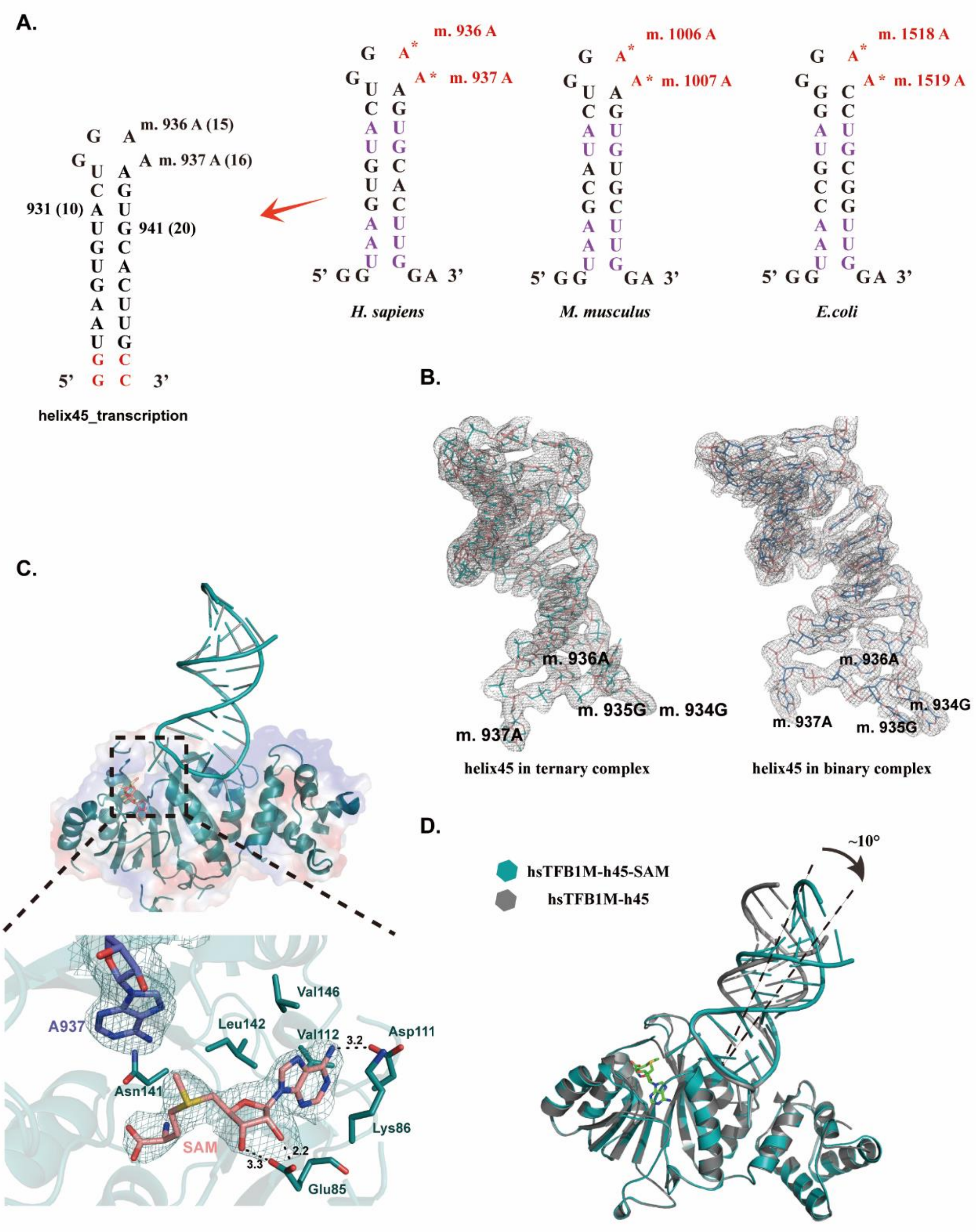

Figure S1. Representation for secondary structures of conserved stem loop at 3' end of rRNA and electron density maps for h45 and SAM. (**A**) Upper right shows the secondary structures of the stem loop showing the sequentially and structurally conserved domains in *H. sapiens*, *M. musculus* and *E. coli*. Nucleotides in purple are conserved regions in stem. The two adenines with asterisks are labeled as the dimethylation modification location. Lower left is presenting the reforming of native helix45 in *H. sapiens* to the sequence for transcription *in vitro* by replacing two cytosines at 3' end. Nucleotides in red (cytosine) are not natural sequences. Numbers in brackets are used in solution structure for convenience. (**B**) Electron density map for h45 in

hsTFB1M-h45-SAM ternary complex structure (left) and hsTFB1M-h45 binary complex (right). The unbiased 2Fo-Fc map (grey) of h45 is contoured at 1.0s. "GGAA" tetraloop are shown as m. 934G, m. 935G, m. 936A and m. 937A. (**C**) Close-up view of the pocket for m. 937A (blue) and SAM (pink). The adjacent interaction residues are colored as deep teal. Hydrogen bonds are marked as black dashed lines. The unbiased 2Fo-Fc map (deep teal) of A937 and SAM is contoured at 1.0s. (**D**) Structural alignment of hsTFB1M-h45 and hsTFB1M-h45-SAM. Color grey presents the structure of hsTFB1M-h45 and deep teal presents hsTFB1M-h45-SAM. 12S rRNA h45 appears an approximate 10° rotation, shown as the black arrow.

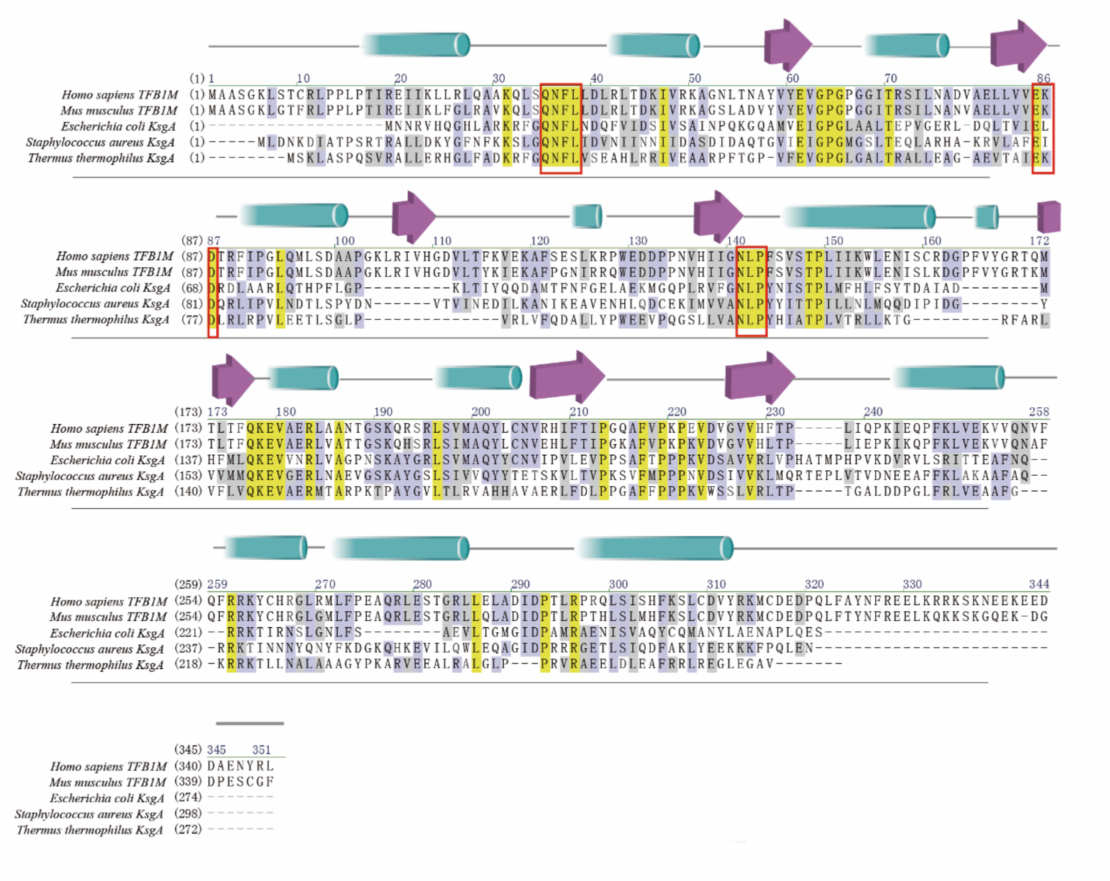

Figure S2. Sequence alignments of *Homo sapiens* TFB1M (NCBI: NP_057104), *Mus musculus* TFB1M (NCBI: NP_666186), *Escherichia coli* KsgA (NCBI: WP_001065373), *Staphylococcus aureus* KsgA (NCBI: WP_000886500) and *Thermus thermophilus* KsgA (NCBI: WP_011227698). Conserved residues of enzyme active center are in red frames.

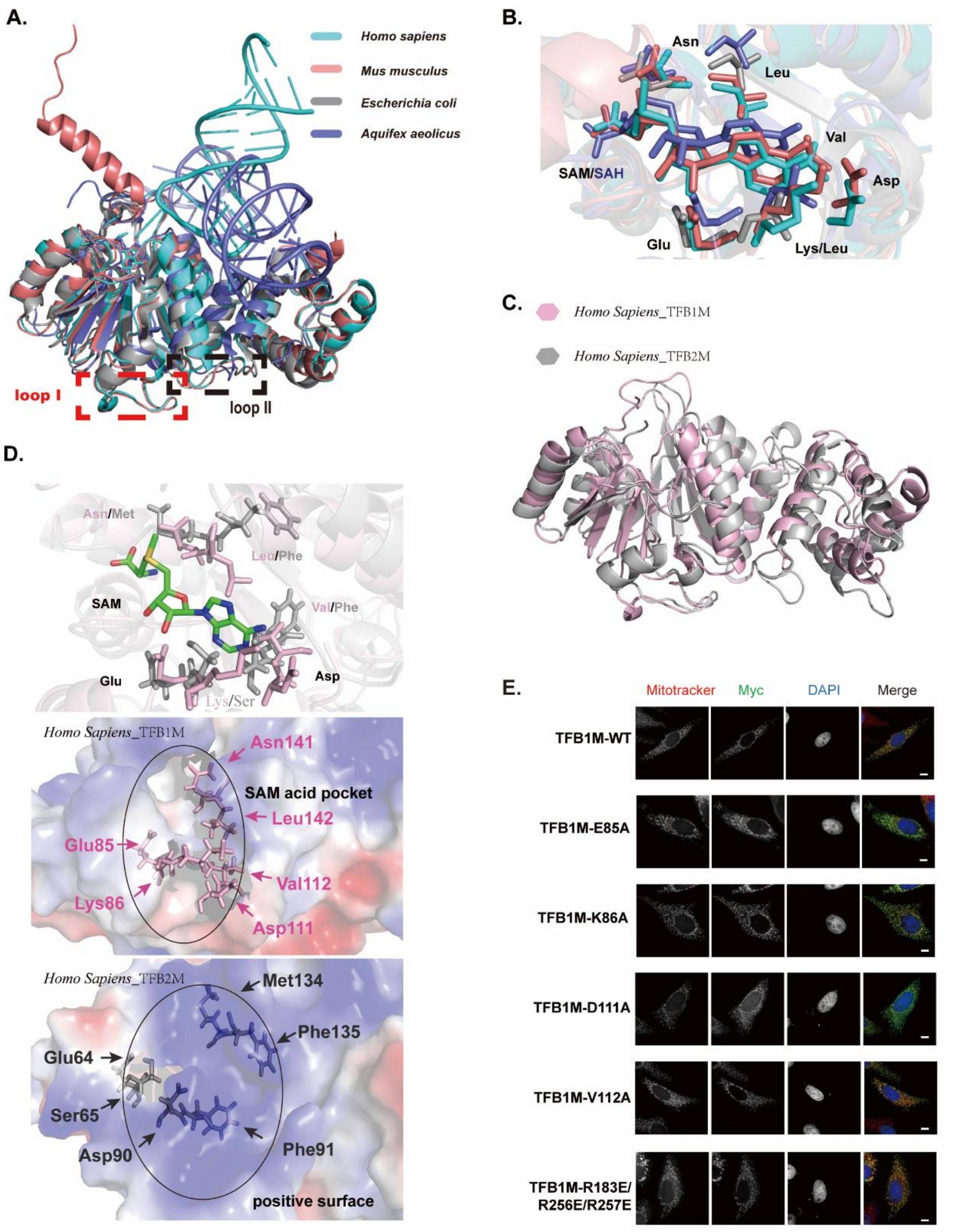

Figure S3. Structural alignments of reported TFB1M or homologous methyltransferases. (**A**) Structural alignment of hsTFB1M-h45-SAM from *Homo sapiens* (cyans; PDB: 6AAX), mmTFB1M-SAM from *Mus musculus* (deepsalmon; PDB ID: 4gc9), KsgA from Escherichia coli (grey; PDB ID: 1qyr) and KsgA-h45-SAH from *Aquifex aeolicus* (slate; PDB ID: 3ftf). (**B**) Structural conservation of SAM/SAH binding pocket. Conserved residues Asn, Leu, Val, Asp, Lys/Leu and Glu are consisted of the binding pocket interacting with the SAM or SAH. (**C**) Structural alignment between hsTFB1M (lightpink) in the ternary complex and TFB2M (grey, PDB: 6ERO). (**D**) Enzyme active center of hsTFB1M and hsTFB2M. Alignment of key residues between these two proteins is showing in left plot. The right pictures are close-up drawing of surface electrostatic charts of

enzyme active center. Colors of hsTFB1M and hsTFB2M are the same with (**C**). The corresponding conserved residues located in active center have been labelled. (**E**) Mitochondrial localization of wild type or mutants of TFB1M in HepG2 cells by immunofluorescence analysis.

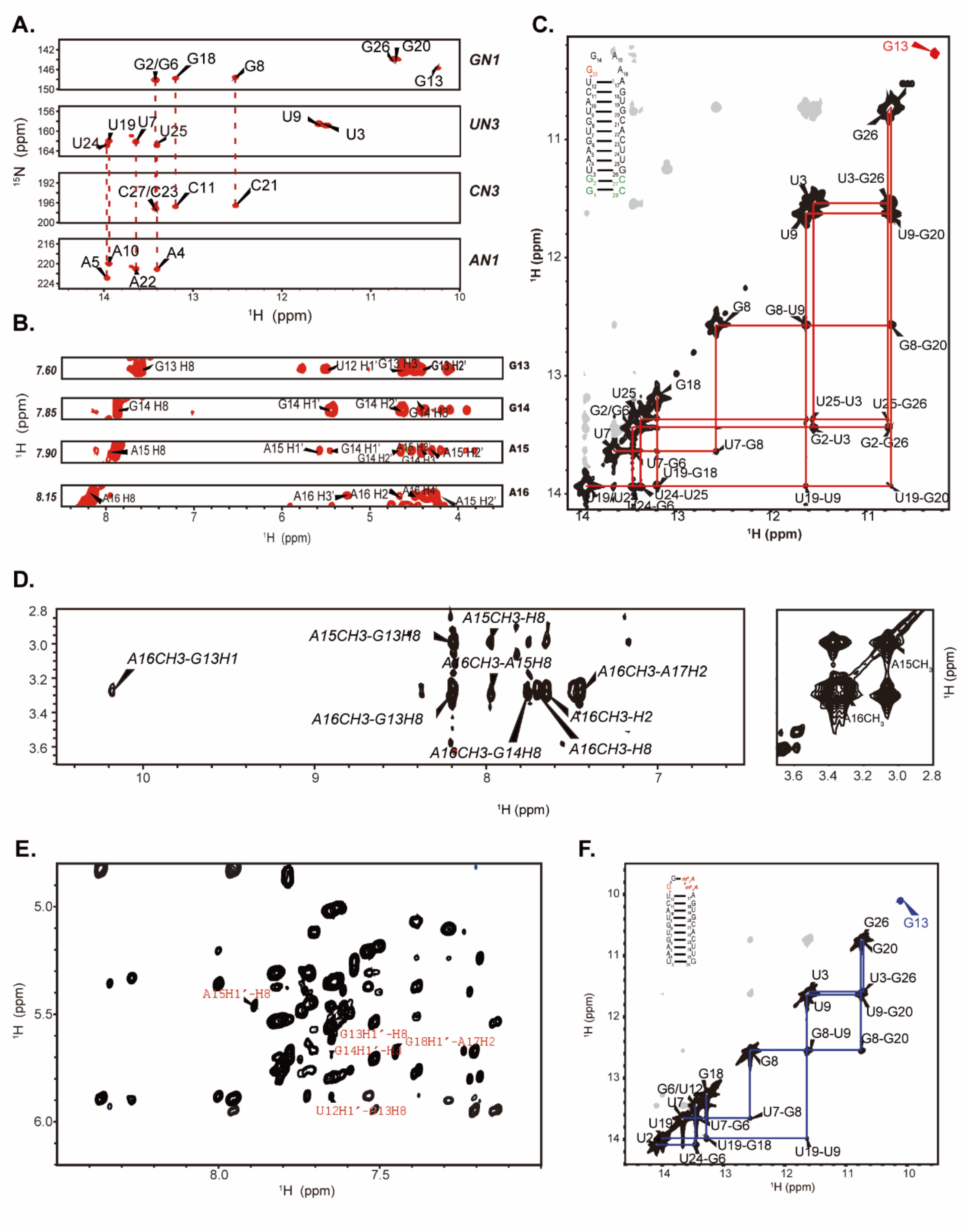

Figure S4. NMR spectra for h45 or m$^{6}_{2}$A-h45. (**A**) Imino proton region of 2D ^{1}H-^{15}N HNN-COSY. The red dash lines are showing the hydrogen-bonded nitrogen atoms of inimo protons in the base pairs. (**B**) Expansion of planes corresponding to C8 regions of the loop "$G_{13}G_{14}A_{15}A_{16}$" collected from 3D ^{1}H-^{13}C NOESY-HSQC spectrum. RNA h45 is uniformly ^{13}C, ^{15}N-labeled in this experiment. (**C**) Imino region of 2D ^{1}H-^{1}H NOESY for h45 employed in H_2O. The secondary structure of h45 is shown on upper left. NOEs among nucleotides for base pairs and adjacent residues are connected by red lines. (**D**) Methyl groups region of 2D ^{1}H-^{1}H NOESY for m$^{6}_{2}$A-h45 in H_2O. Only one diagonal peak for CH_3 of A15, but two for A16 (right). The cross-peaks for CH_3 and H8/H2/H1 are presented on left. (**E**) Portion of 2D ^{1}H-^{1}H NOESY for unlabeled m$^{6}_{2}$A-h45. The cross-peaks

between aromatic H8 protons and ribose H1' protons of the nucleotides in or near loop are labeled. (**F**) Imino region of 2D ^{1}H-^{1}H NOESY for $m^{6}_{2}A$-h45 in H_2O.

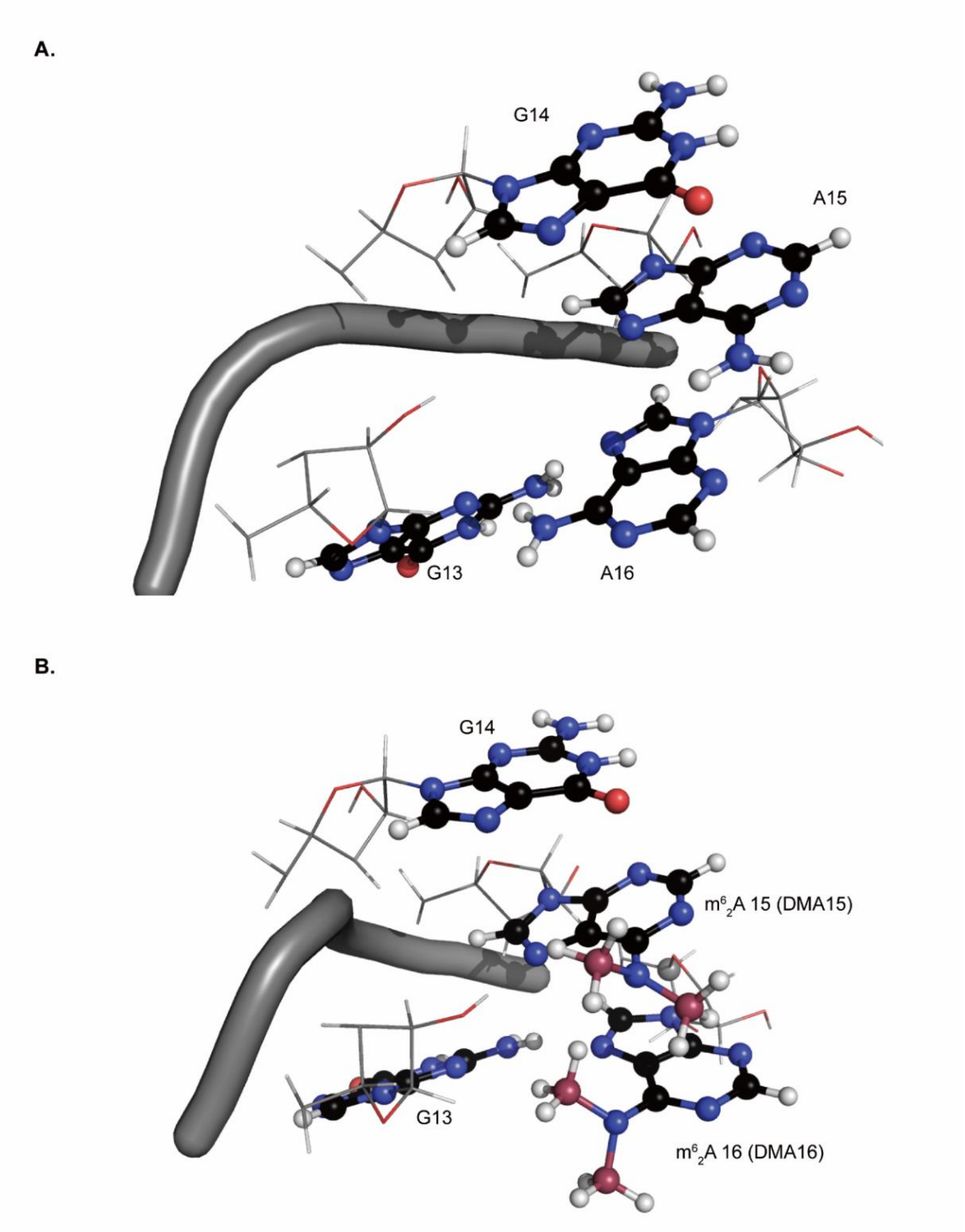

Figure S5. Local conformations of the loop regions of h45 and m$^6{}_2$A-h45. (**A**) Ribbon-and-sphere model for the "GGAA" tetraloop of h45. Carbon, nitrogen, hydrogen and oxygen atoms are colored in black, blue, white and red, respectively. (**B**) Ribbon-and-sphere model for the "GGm$^6{}_2$Am$^6{}_2$A" tetraloop of m$^6{}_2$A-h45. Methyl carbons are colored in raspberry. DMA is the name given to m$^6{}_2$A in the CYANA library.

Table S1. Oligonucleotides sequece for RNA and primer

Name	sequence
h45 RNA	5'- GG UAAGUGUACUGGAAAGUGCACUUG CC - 3'
h45_primer	5'- GGCAAGTGCACTTTCCAGTACACTTACCTATAGTGAGTCGTATTAATTTC- 3'
$m^6{}_2A$-h45 RNA	5'- UAAGUGUACUGG $m^6{}_2A$(DMA) $m^6{}_2A$(DMA) AGUGCACUUG - 3'
T7 promoter_primer	5'- GAAATTAATACGACTCACTATAG -3'
hsTFB1M_Forward primer	5'- CGCGGATCCATGCAAGCAGCGAAGCAGCTATCACAG -3'
hsTFB1M_Reverse forward primer	5'- CCGCTCGAGCTAGAGTCTGTAATTCTCTGCGTCATC -3'
5’-Cy5_DNA primer	5'- CTGGTTCGTCCAAGTG - 3'
5’-FAM_h45 RNA (WT)	5'- GG UAAGUGUACUGGAAAGUGCACUUG CC - 3'
sh3 (shRNA 3)	CGCAGAGAATTACAGACTCTA
sh4 (shRNA 4)	CCACGATTCGAGAAATCATTA
sh3' UTR (shRNA 3' UTR)	GGATCTGCGCCTTGAGCAAAG

Table S2. NMR restraints and structure statistics for the 20 lowest energy structures of h45 and m^6_2A-h45

**Cyana**[1]	**h45**	**m^6_2A-h45**
NOE-derived restraints	211	166
Intraresidue	84	63
Interresidue	127	103
H-bond restraints	60	45
NOE restraints/residue	7.54	6.92
Total restraints/residue	9.68	8.79
Upper distance viol. (Å)	0.0015±0.0007	0.0032±0.0013
Lower distance viol. (Å)	0.0015±0.0006	0.0013±0.0010
Sum VDW viol. (Å)	0.8±0.0	0.6±0.2
RMSD (Å)[2]	1.23±0.40	0.92±0.26
RMSD stem (Å) (G1-U12, A17-C28/U3-U12, A17-G26)	0.63±0.20	0.72±0.28
**Amber**[3]	**h45**	**m^6_2A-h45**
Amber energy (kcal mol-1)	-6320.50 (17.79)	-5056.01 (10.28)
Distance (kcal mol-1)	3.022 (1.16)	2.061 (0.97)
Torsion (kcal mol-1)	0.229 (0.02)	0.175 (0.03)
RMSD (Å)[4]	0.956	0.648
RMSD stem (Å) (G1-U12, A17-C28/U3-U12, A17-G26)	0.118	0.521
**MolProbity**[5]	**h45**	**m^6_2A-h45**
Clashscore	0.46 (0.68)	0.14 (0.43)
Probably wrong sugar pucker (%)	0.2 (0.41)	0 (0)
Bad backbone conformation (%)	1.3 (1.03)	0.05 (0.22)
Bad bonds (%)	2 (0)	5 (0)
Bad angles (%)	0 (0)	0 (0)
PDB ID	6AAS	6AAU
BMRB	36202	27552

[1] Statistics for the 20 structures with lowest target function.

[2] Mean standard deviation for all heavy atoms, relative to mean coordinates, calculated with Cyana.

[3] Statistics for the 20 lowest energy structures.

[4] Mean standard deviation for all heavy atoms, relative to the average structure, calculated using suppose over the residues listed in parentheses.

[5] The 20 amber-refined structures were evaluated using the MolProbity webserver.

Table S3. Parameters for crystal of hsTFB1M-h45-SAM.

PDB ID	6AJK	6AAX
**Data collection**	**hsTFB1M-h45**	**hsTFB1M-h45-SAM**
Wavelength (Å)	0.9790	0.9790
Space group	*P 4₃22*	*P 6₁22*
Cell dimensions		
a, b, c (Å)	122.564, 122.564, 73.067	104.12, 104.12, 449.38
α, β, γ(°)	90, 90, 90	90, 90, 120
Resolution range (Å)	35.66 - 3.001 (3.108 - 3.001)	38.63 - 2.99 (3.10 - 2.99)
R_{pim} (%)	10.1（32.2）	4.20 (35.3)
I/σI	4.67（1.50）	31.00 (2.75)
Completeness (%)	98.15 (99.56)	99.82 (99.56)
Redundancy	3.5（3.5）	42.0(43.0)
Wilson B factor (Å^2)	30.23	64.05
**Refinement**	**hsTFB1M-h45**	**hsTFB1M-h45-SAM**
Resolution (Å)		
No. reflections	11431/551	30268/1473
R_{work}/R_{free} (%)	19.65/24.10	22.37/26.66
No. atoms		
Protein	2343	2261
RNA	601	601
B-factors (Å^2)		
Protein	23.86	57.93
RNA	28.39	74.51
R. m. s. deviations		
Bond lengths (Å)	0.007	0.009
Bond angles (°)	0.97	1.230
**Ramachandran analysis**	**hsTFB1M-h45**	**hsTFB1M-h45-SAM**
Favored regions (%)	94.85	91.52
Allowed regions (%)	4.81	7.79
Outlier regions (%)	0.34	0.69
